# Prevalence of sustainable and unsustainable use of wild species inferred from the IUCN Red List of Threatened Species

**DOI:** 10.1111/cobi.13844

**Published:** 2021-11-17

**Authors:** Sophie M. E. Marsh, Michael Hoffmann, Neil D. Burgess, Thomas M. Brooks, Daniel W. S. Challender, Patricia J. Cremona, Craig Hilton‐Taylor, Flore Lafaye de Micheaux, Gabriela Lichtenstein, Dilys Roe, Monika Böhm

**Affiliations:** ^1^ Centre for Biodiversity and Environment Research, Department of Genetics, Evolution and Environment University College London London UK; ^2^ Conservation and Policy Zoological Society of London, Regent's Park London UK; ^3^ UNEP‐WCMC Cambridge UK; ^4^ CMEC, GLOBE Institute University of Copenhagen Copenhagen Denmark; ^5^ International Union for Conservation of Nature Gland Switzerland; ^6^ World Agroforestry Center (ICRAF) University of the Philippines Los Baños The Philippines; ^7^ Institute for Marine and Antarctic Studies University of Tasmania Hobart Tasmania Australia; ^8^ Department of Zoology University of Oxford Oxford UK; ^9^ International Union for Conservation of Nature Cambridge UK; ^10^ Institute of Geography and Sustainability University of Lausanne Lausanne Switzerland; ^11^ French Institute of Pondicherry Pondicherry India; ^12^ Instituto Nacional de Antropología y Pensamiento Latinoamericano (INAPL)/CONICET Buenos Aires Argentina; ^13^ International Institute for Environment and Development (IIED) and IUCN Sustainable Use and Livelihoods Specialist Group (SULi) London UK; ^14^ Institute of Zoology Zoological Society of London London UK

**Keywords:** CITES, conservation action, Convention on Biological Diversity, exploitation, IPBES, sustainable use, unsustainable uses, wildlife, acción de conservación, CITES, Convenio sobre la Diversidad Biológica, explotación, fauna silvestre, IPBES, usos no sustentables, uso sustentable

## Abstract

Unsustainable exploitation of wild species represents a serious threat to biodiversity and to the livelihoods of local communities and Indigenous peoples. However, managed, sustainable use has the potential to forestall extinctions, aid recovery, and meet human needs. We analyzed species‐level data for 30,923 species from 13 taxonomic groups on the International Union for Conservation of Nature Red List of Threatened Species to investigate patterns of intentional biological resource use. Forty percent of species (10,098 of 25,009 species from 10 data‐sufficient taxonomic groups) were used. The main purposes of use were pets, display animals, horticulture, and human consumption. Intentional use is currently contributing to elevated extinction risk for 28–29% of threatened or near threatened (NT) species (2752–2848 of 9753 species). Intentional use also affected 16% of all species used (1597–1631 of 10,098). However, 72% of used species (7291 of 10,098) were least concern, of which nearly half (3469) also had stable or improving population trends. The remainder were not documented as threatened by biological resource use, including at least 172 threatened or NT species with stable or improving populations. About one‐third of species that had use documented as a threat had no targeted species management actions to directly address this threat. To improve use‐related red‐list data, we suggest small amendments to the relevant classification schemes and required supporting documentation. Our findings on the prevalence of sustainable and unsustainable use, and variation across taxa, can inform international policy making, including the Intergovernmental Science‐Policy Platform on Biodiversity and Ecosystem Services, the Convention on Biological Diversity, and the Convention on International Trade in Endangered Species.

## Abstract 2

*Article impact statement*: Of 10,098 species on the IUCN Red List that are harvested, use is likely biologically unsustainable for 16% and sustainable for 34%.

## Abstract (es)

Predominio del Uso Sustentable y No Sustentable de Especies Silvestres Inferido a partir de la Lista Roja de Especies Amenazadas de la UICN

**Resumen:**

La explotación insostenible de especies silvestres representa una verdadera amenaza para la biodiversidad y el sustento de las comunidades locales y los pueblos indígenas. Sin embargo, el uso sostenible gestionado tiene el potencial para prevenir extinciones, auxiliar en la recuperación y satisfacer las necesidades humanas. Analizamos los datos a nivel de especie correspondientes a 30,923 especies de 13 grupos taxonómicos localizados en la Lista Roja de Especies Amenazadas de la Unión Internacional para la Conservación de la Naturaleza (UICN) para investigar los patrones del uso intencional de recursos biológicos. Usamos el 40% de las especies analizadas (10,098 de 25,009 especies pertenecientes a diez grupos taxonómicos con suficiente información). Los principales motivos para el uso de vida silvestre fueron como mascotas, animales de exhibición, horticultura y consumo humano. El uso intencional está actualmente contribuyendo a un riesgo elevado de extinción para 28 ‐ 29% de las especies amenazadas o casi amenazadas (NT) (2,752 ‐ 2,848 de 9,753 especies). El uso intencional también afectó al 16% de todas las especies utilizadas (1,597 ‐ 1,631 de 10,098). Sin embargo, el 72% de las especies utilizadas (7,291 de 10, 098) pertenecen a la categoría de preocupación menor, de las cuales casi la mitad (3,469) también contaban con tendencias poblacionales estables o de mejoría. Las especies restantes no estaban documentadas como amenazadas por el uso de recursos biológicos, incluyendo al menos 172 especies amenazadas o NT con poblaciones estables o en aumento. Casi un tercio de las especies que tienen documentado el uso como una amenaza no cuentan con acciones de manejo para abordar directamente esta amenaza. Para mejorar la información de la lista roja relacionada con el uso, sugerimos pequeñas modificaciones a los esquemas relevantes de clasificación y la documentación de apoyo requerida. Nuestros descubrimientos sobre el predominio del uso sustentable y no sustentable, y la variación entre taxones, puede orientar la formulación de políticas internacionales, incluyendo a la Plataforma Intergubernamental de Políticas Científicas sobre Biodiversidad y Servicios Ecosistémicos, el Convenio sobre la Diversidad Biológica y la Convención sobre el Comercio Internacional de Especies Amenazadas.

## INTRODUCTION

It is critical to understand and manage the impacts of threats related to the use of wild species to ensure their survival while continuing to support global demand for biological resources. Overexploitation is among the predominant threats to many species (di Minin et al., 2019; Maxwell et al., 2016) and the primary threat to aquatic species (IPBES, 2019). Nonetheless, billions of people rely on wild species, including plants, animals, and fungi, for food, medicines, construction materials, and other uses (Nasi et al., 2011; Thilsted et al., 2016). The use of wild species underpins the livelihoods of millions of people and has cultural, religious, and recreational value. These values in turn provide a local incentive for the conservation of species. The tension between overexploitation as a major driver of biodiversity loss, and humanity's reliance on wild species for many different needs creates a conundrum. How can biological resource use (BRU) be managed in sustainable ways that help meet human needs and incentivize conservation, rather than further driving species to extinction?

The use of wild species can be sustainable given adequate management (Austin & Corey, 2012; Lichtenstein, 2010). The concept of sustainable use is embedded in many international and national regulatory and policy frameworks as a conservation tool and as a way to promote human development and ensure availability of natural resources for future generations. It is one of the three primary objectives of the Convention on Biological Diversity (CBD) and is explicitly stated in the UN Sustainable Development Goals. Nonetheless, sustainable use as a practice remains polarizing (Challender & MacMillan, 2019; Hutton & Leader‐Williams, 2003), especially consumptive use of animals (involving the removal of either live or dead individuals), and there is limited consensus regarding the effectiveness of different approaches. This issue is exacerbated by concerns that inaction or ineffective sustainable use policies could rapidly imperil many already threatened species (Auliya et al., 2016). Conversely, actions to prevent or reduce use could have negative consequences (Bonwitt et al., 2018; Cooney & Jepson, 2006) for species and people who depend on their use.

The discourse around sustainable use is further hampered by knowledge gaps. Although there is a good body of research on how use and trade drive declines and imperil species, understanding of different patterns of use within species is limited. The same can be said for the degree to which species currently affected by overexploitation are receiving appropriate conservation actions, or where, and under what circumstances, trade is taking place at sustainable levels without threatening species (Morton et al., 2021). Several data sources that can provide insights into sustainable versus unsustainable use exist, but may be limited in geographical or taxonomic scope. For example, McRae et al. (2020) used a global data set of over 11,000 population time series from the Living Planet Database to derive trends in utilized versus unutilized vertebrates and to assess whether management makes a measurable difference to wildlife population trends for utilized species. Although the underlying data are population‐specific and provide a higher resolution than species‐level databases, the taxonomic scope is limited to vertebrates only. Other data sets can provide taxon‐ and region‐specific insight into the use of species, including Prota4U for plants (https://www.prota4u.org/) and national red lists. However, they are not directly comparable for global analyses, not least because they use different taxonomies and have different protocols for capturing information on use.

The International Union for Conservation of Nature (IUCN) Red List of Threatened Species (henceforth Red List) provides global data for a wide range of taxa that can help managers and policy makers understand and deliver targeted action to address threats to biodiversity. The role of the Red List in supporting and influencing global policy instruments is well established, for instance, in tracking progress toward globally agreed targets, such as the CBD Aichi Targets (SCBD, 2014), new targets under discussion in the post‐2020 Global Biodiversity Framework, and Sustainable Development Goals (Brooks et al., 2015). The Red List also provides key data and trends that inform processes in the Convention on International Trade in Endangered Species of Fauna and Flora (CITES; Challender et al., 2019) and the Intergovernmental Science‐Policy Platform on Biodiversity and Ecosystem Services (IPBES; Brooks et al., 2016; IPBES, 2019).

Individual red‐list assessments are carried out by thousands of scientific experts in accordance with a system of objective, quantitative categories and criteria that rank a species’ extinction risk from least concern (LC) to extinct in the wild (EW), or extinct (EX). A species is considered threatened if it is assessed as vulnerable (VU), endangered (EN), or critically endangered (CR) (IUCN, 2012). Assessments follow well‐defined guidelines with an independent process for review (Collen et al., 2016) and are underpinned by ancillary data on distribution, population size and trend, habitat preferences, threats, and conservation actions in place or needed. Much of this information is recorded in standardized classification schemes that enable comparative analyses across taxa.

Previous analyses of BRU based on red‐list data have focused on individual taxonomic groups (e.g., birds [Butchart, 2008], cacti [Goettsch et al., 2015]) or particular dimensions of use (e.g., traded vertebrates [Scheffers et al., 2019], spatial concentrations of unsustainable use [di Minin et al., 2019]). We substantially advance previous analyses of red‐list data by investigating all patterns of intentional BRU across a broad suite of species assessed by IUCN. We asked what are the main purposes of use of wild animal and plant species; for which species are current levels of use having a negative impact on species extinction risk and hence likely to be biologically unsustainable; for which species are current levels of use not having a negative impact on species extinction risk and hence likely to be biologically sustainable; and for which utilized species are conservation actions currently in place that directly address impacts from current levels of use. We sought to provide a framework for future replications of use analyses (e.g., to track trends over time) and suggestions for improving the quality of use‐related data in future IUCN assessments.

## METHODS

### Species data

We collated species‐level data for 13 taxonomic groups that have been comprehensively assessed by IUCN (version 2020–1). The IUCN defines comprehensively assessed groups as taxonomic groups that include at least 150 species, of which >80% have been assessed (IUCN, 2020). Noncomprehensively assessed groups may primarily focus on species that are likely threatened or likely used by people or may have a regional focus. Excluding these groups avoids introducing additional biases into our analysis, for instance, threat processes that are not evenly distributed geographically (Miqueleiz et al., 2020).

We classified the 13 taxonomic groups into six primarily aquatic (freshwater, marine, or both) and seven primarily terrestrial groups (including amphibians, among which ∼30% are documented as terrestrial only, the remainder as both terrestrial and freshwater). We excluded all species listed as EX or EW, because neither can be used in the wild, and data deficient (DD), because the impact of use on their extinction risk is unknown. This restricted our analyses to LC, near threatened (NT), and threatened species only (hereafter extant, data‐sufficient species). This data set comprised 30,923 species made up of 6603 primarily aquatic species and 24,320 primarily terrestrial species (Table 1 & Appendix S1).

**TABLE 1:** Species groups comprehensively assessed by the International Union for Conservation of Nature and subsets included in analyses of the main purposes of use of wild animal and plant species, use that is likely biologically sustainable or unsustainable, and the conservation actions in place to address the impacts of use and their respective sample sizes

			Documentation of use^b^							Documentation of targeted species management actions **^d^			
	Species data^a^		Use*^e^			Use**^f^		Documentation of intentional biological resource use (BRU)^c^			Intentional BRU^g^		
Taxonomic group^h^	n*^i^	n**^j^	All	LC^k^	LC and not declining^l^	All	Not declining and no BRU^m^	Use*^n^	Minimum – Maximum **^o^	All	Minimum	Targeted species management, or unknown^p^	No targeted action^q^
Aquatic	6603	1589	2674	2234	495	577	7–44	518–519	946–963	277	236	158	81
selected bony fishes	2649	257	1386	1249	413	137	3–13	120	134–140	95	78	74	1
crustaceans	1749	552	263	200	48	63	0–17	37–38	58–69	46	24	23	1
cartilaginous fishes	686	318	N/A	N/A	N/A	N/A	N/A	N/A	314–314	136	134	55	79
corals	643	388	481	170	6	311	0	311	388–388	0	0	0	0
cone snails	545	67	544	478	28	66	4–14	50	50–50	0	0	0	0
cephalopods	331	7	N/A	N/A	N/A	N/A	N/A	N/A	2–2	0	0	0	0
Terrestrial	24,320	8164	7424	5057	2974	2230	165–242	1079–1112	1806–1885	4450	1363	827–831	125
birds	10,930	2503	4988	3852	2120	1136	117–144	366	406–407	2491	406	171	8
amphibians	5406	2577	576	341	160	235	6–8	176	195–195	91	39	35	2
mammals	4897	1591	N/A	N/A	N/A	N/A	N/A	N/A	615–618	717	441	404–405	25
selected dicots	1898	791	1094	653	462	441	16–37	287–301	311–361	552	231	192–195	27
conifers	602	304	458	243	169	215	19–35	103–120	106–126	251	89	13	63
cycads	300	255	177	29	20	148	4–5	125–127	147–152	229	134	134	0
selected reptiles	287	143	131	76	43	55	3–13	22	26–26	119	23	22	0
Total	30,923^r^	9753	10,098	7291	3469	2807	172–286	1597–1631	2752–2848	2848	1599	985–989	206
Outdated assessments (<2010)^s^	4089 (13%)	2063 (21%)	606 (6%)	258 (4%)	104 (3%)	348 (12%)	6–10 (3%)	318 (19–20%)	690–693 (24‐25%)	196 (7%)	152 (10%)	146 (15%)	6 (3%)
Analysis^t^	1	2b	1, 2a, 3a,b	3a	3b	3c	3c	2a	2b	mentioned in text	4a	4a	4b
Figure^u^	1	2	3	3	3	3			2, Appendix S13		4, Appendix S15	4, Appendix S16	Appendix S17

^a^ Number of species selected for analysis in this study.

^b^ Number of species documented as being used based on the International Union for Conservation of Nature (IUCN)’s use and trade classification scheme, excluding uses for ex situ propagation, other, or unknown purposes (Appendix S3). Cephalopods, cartilaginous fishes, and mammals were excluded due to insufficient data (Appendix S5).

^c^ Number of species negatively affected by biological resource use (BRU) based on the IUCN's threats classification scheme (Appendix S2).

^d^ Double asterisk denotes extant, data‐sufficient species assessed as near threatened (NT) or threatened. Number of species with documentation of either species’ harvest management plan or international trade control, recorded as yes, no, or unknown in the IUCN's conservation actions in place classification scheme.

^e^ Single asterisk denotes extant, data‐sufficient species.

^f^ Double asterisk denotes extant, data‐sufficient species assessed as NT or threatened.

^g^ Minimum number (see Methods) of species negatively affected by intentional BRU based on the IUCN's threats classification scheme.

^h^ Taxonomic groups included in this study, classed as aquatic for primarily aquatic groups and terrestrial for primarily terrestrial groups. Bony fishes, dicotyledons (dicots), and reptiles include selected higher‐level taxa, see Appendix S1 for detailed listing of taxonomic groups.

^i^ Number of extant, data‐sufficient species included in this study.

^j^ Number of extant, data‐sufficient species assessed as NT or threatened included in this study.

^k^ Number of species assessed as least concern (LC).

^l^ Number of species assessed as LC that had stable or increasing population trends at the time of red‐list assessment.

^m^ Number of NT or threatened species that had stable or increasing population trends at the time of red‐list assessment, and are not affected by major intentional BRU.

^n^ Number of extant, data‐sufficient species that are used based on the IUCN's use and trade classification scheme.

^o^ Double asterisk denotes extant, data‐sufficient species assessed as NT or threatened. Range of minimum to maximum number (see Methods) of species that are negatively affected by BRU based on the IUCN's threats classification scheme.

^p^ Number of species that receive species’ harvest management or international trade controls (documented as yes in the IUCN's conservation actions in place classification scheme), or may receive either type of management (documented as unknown in the IUCN's conservation actions in place classification scheme; included in the higher range number for mammals and selected dicots).

^q^ Number of species not receiving species’ harvest management or international trade controls (documented as no under the IUCN's conservation actions in place classification scheme).

^r^ The total number of extant, data‐sufficient species is 25,009 when excluding species groups without adequate documentation of use based on the IUCN's use and trade classification scheme.

^s^ Number and percentage of species in each column that have not been reassessed since 2009.

^t^ Analysis corresponding to each column, as described in methods and summarized in Appendix S12.

^u^ Figure or figures presenting the results of each analysis.

### Red‐List data used

For each species, we downloaded the following data from the Red List: category, current population trend, threats, use and trade, and conservation actions in place. Information on the latter three is recorded for each species based on IUCN's standardized classification schemes, which provide a harmonized typology for recording relevant attributes (Salafsky et al., 2008). Documenting population trends at time of assessment is mandatory for all IUCN species assessments, and trends are presented as stable, decreasing, increasing, or unknown. Use of species is captured in two distinct ways: as a threat under IUCN's threats classification scheme (class 5, BRU [Salafsky et al., 2008; Appendix S2]) and under the use and trade classification scheme, which explicitly does not associate the use with a threat (Appendix S3). The threat information says whether species are negatively affected by use, whereas use and trade information documents the purposes of use regardless of whether it represents a threat. Although the recording of major threats affecting a species is mandatory for EX, EW, threatened, and NT species (IUCN, 2016), recording of use and trade is only recommended (i.e., strongly encouraged, but not mandatory) for species listed in any other category, and may thus not be consistently documented across all species on the Red List. Conservation actions are recorded in the conservation actions in place classification scheme and conservation actions needed classification scheme. In both schemes, this information is also only recommended documentation and thus may not be consistently recorded (Luther et al., 2016). For the purpose of this study, we analyzed only the conservation actions in place classification scheme.

### Main purposes of use of wild animal and plant species

We investigated the prevalence of different purposes of use based on the information recorded in the use and trade classification scheme, excluding records for establishing ex situ production (use code 16), other (17), and unknown (18). For this analysis, we limited our data set to 10 taxonomic groups that had adequate recording of use and trade (details on how adequate recording of use was defined in Appendix S4); thus, cartilaginous fishes and cephalopods from the aquatic species group and mammals (a high‐profile group when it comes to discussion of use) from the terrestrial group were excluded. For each taxonomic group, we calculated the total number of species recorded as being used for at least one purpose in the use and trade classification scheme. We then summarized results as the percentage of species recorded for different types of use on the Red List out of all extant, data‐sufficient species (totaling 25,009 species in 10 taxonomic groups) (Table 1) (full list of selected groups in Appendix S5).

### Wild species for which intentional use has a negative impact on extinction risk

We considered use biologically unsustainable when it is likely contributing to species extinction risk. To identify such cases, we analyzed the proportion of species for which BRU was documented as a major threat, based on IUCN's threat classification scheme, from among all species with at least one purpose of use coded (from among the 10 taxonomic groups with adequate data) and all NT and threatened species (from all 13 taxonomic groups comprehensively assessed) (Table 1). Because not all types of BRU intentionally target a species, we developed a decision tree for removing threat types that are not relevant to an analysis of direct, intentional use of species (Appendix S6). In some cases, we were unable to determine whether use was intentional. This uncertainty is presented as a range: the minimum proportion included all species with threats that could be conclusively determined as intentional and the maximum proportion additionally included species for which use motivation was unknown or unrecorded that may represent further cases of intentional BRU. For groups in which motivation for use of species was unknown or unrecorded, we present only the minimum (Appendix S7).

We included BRU as a threat if it had a medium to high impact on species extinction risk. The IUCN uses a scoring system to estimate threat impact that is based on timing, scope, and severity of the threat. This information is used to create an overall threat impact score. Threat timing is recommended information in IUCN assessments (i.e., strongly encouraged but not essential to publication), whereas severity and scope are discretionary and often not included. Thus, we amended the threat impact score categorization to help exclude threats that were unlikely to be major (Appendix S8).

### Wild species for which intentional use does not have a negative impact on extinction risk

We considered use likely biologically sustainable when it was unlikely to have an adverse impact on species extinction risk. Due to data constraints, we could only derive a minimum estimate by determining the number of species recorded as subject to some form of use or trade that were LC; LC and not declining (i.e., stable or increasing current population trends); and threatened or NT with no intentional use or intentional use documented as a minor threat only and stable or increasing population trends (Table 1 & Appendix S9). We recognize the limitations of using overall current population trends. However, our first approach ties population trend to extinction risk (specifically, if a species is LC, then intentional use or trade, or indeed any threat, cannot, by definition, be a major threat), whereas our second approach is explicitly tied to threatened or NT species that do not have use or trade documented as a major threat; as such, this cannot be a primary factor driving elevated extinction risk. We confined analyses to those 10 taxonomic groups for which use and trade information was adequate.

### Conservation actions in place or lacking for utilized wild species

To understand the current level of conservation actions in place to respond to overexploitation, we extracted all NT and threatened species documented as receiving targeted species management actions, as recorded via the conservation actions in place classification scheme. Specifically, we selected species that had a harvest management plan and species subject to international management or trade controls (e.g., CITES or U.S. Endangered Species Act listing, regional fisheries agreements). We then determined the number of species negatively affected by BRU and recorded as receiving either one or both of these actions or not receiving either of these actions (Table 1).

Appendix S10 has a structured outline of our methods, and Appendix S11 has a glossary of terms.

### Outdated assessments

Thirteen percent of our complete data set of extant, data‐sufficient species has not been reassessed in the last 10 years (considered out‐of‐date by IUCN standards); this is 22% of threatened or NT species (Table 1). This percentage is lower than the overall percentage of species with outdated assessments (version 2020–3) on the Red List (23.8%). Among those species for which documentation of use is available, only 6% of species have outdated assessments (Appendix S12). Of these, two taxonomic groups have a higher proportion of species with outdated assessments than average (corals 62% and amphibians 44%). Older assessments may not account properly for current patterns of use (equally, their extinction risk categories, threats, current population trends, and other supporting information might have changed). Nonetheless, we considered it appropriate to include these species because these data remain the best available in the context of our global analysis, and both corals and amphibians were compiled as part of major global assessment processes that did include explicit documentation of use. However, to assess whether the inclusion of outdated assessments skewed our results, we ran a chi‐square test in which we excluded outdated assessments from our data. Specifically, we analyzed whether the distribution of species for which use was likely biologically sustainable (LC or threatened or NT and not declining and not affected by intentional BRU) versus those for which use was likely biologically unsustainable (all used species affected by intentional BRU) was significantly different when discounting outdated assessments.

## RESULTS

### Main purposes of use of wild animal and plant species

Among the 10 taxonomic groups with adequate information, the proportion of extant, data‐sufficient species documented as having at least one purpose of use coded ranged from 15% (crustaceans) to nearly 100% (cone snails, 544 of 545 species) among aquatic groups and from 11% (amphibians) to 76% (conifers) among terrestrial groups. Across the 25,009 species in these 10 groups, 10,098 (40%) had some purpose of use documented (Table 1).

**FIGURE 1:**
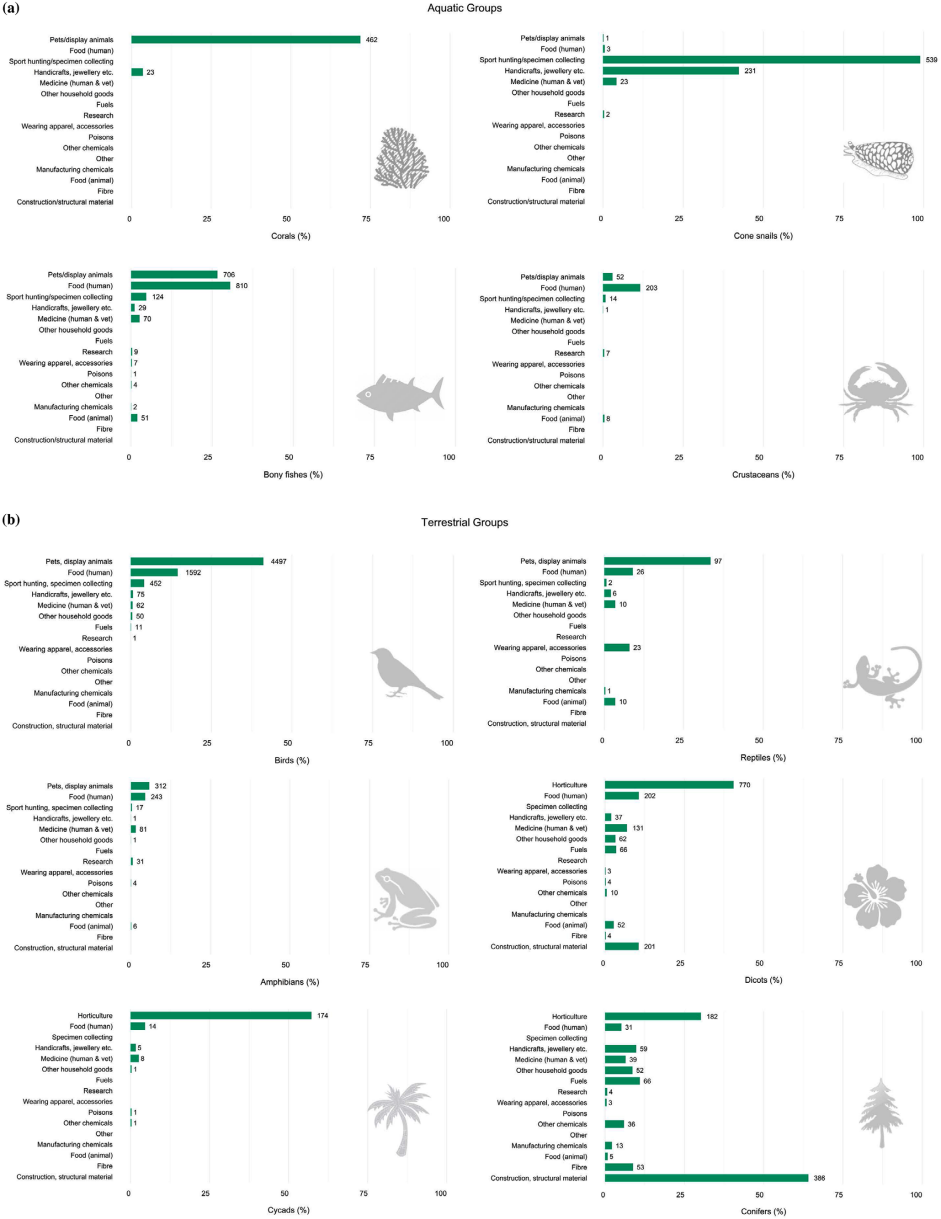
Percentage of extant, data‐sufficient species in (a) aquatic and (b) terrestrial taxonomic groups recorded for different types of use on the International Union for Conservation of Nature Red List. Percentages are out of total extant, data‐sufficient species (Table 1). Numbers next to bars are total number of species recorded for each type of use. Most species are subject to more than one type of use. Bony fishes, dicotyledons (dicots), and reptiles include selected higher‐level taxa (Appendix S1)

In the aquatic groups, the top purposes of use were human food (selected bony fishes and crustaceans), specimen collection (cone snails), and pets and display animals (corals and selected bony fishes) (Figure 1). Additional purposes of use were handicrafts and jewelry (cone snails and corals) and medical purposes (cone snails). For terrestrial animal groups, the two most prevalent uses were pets or display animals and human consumption (birds and herptiles). This was followed by sport hunting and specimen collecting for birds, medicinal purposes for amphibians, and apparel or accessories for selected reptiles. For plant taxonomic groups, the predominant uses were building materials (conifers) and horticulture (all three groups). Overall, plant groups had more purposes than animal groups, including human and animal food, medicine, household goods, and handicrafts or jewelry, fuels, and chemicals.

### Wild species for which intentional use has a negative impact on extinction risk

Considering all 10,098 species for which some purpose of use was documented in the 10 taxonomic groups with adequate information, a sixth had intentional BRU as a threat (1597–1631 species, including those with motivation unknown [16%]). Moreover, more than one‐quarter of all NT and threatened species across all 13 comprehensively assessed taxa (9753 species) had intentional BRU documented as a threat (minimum 2752 species [28%]; maximum 2848 [29%]). Excluding outdated assessments (2063 species) yielded a similar proportion (27–28%).

**FIGURE 2:**
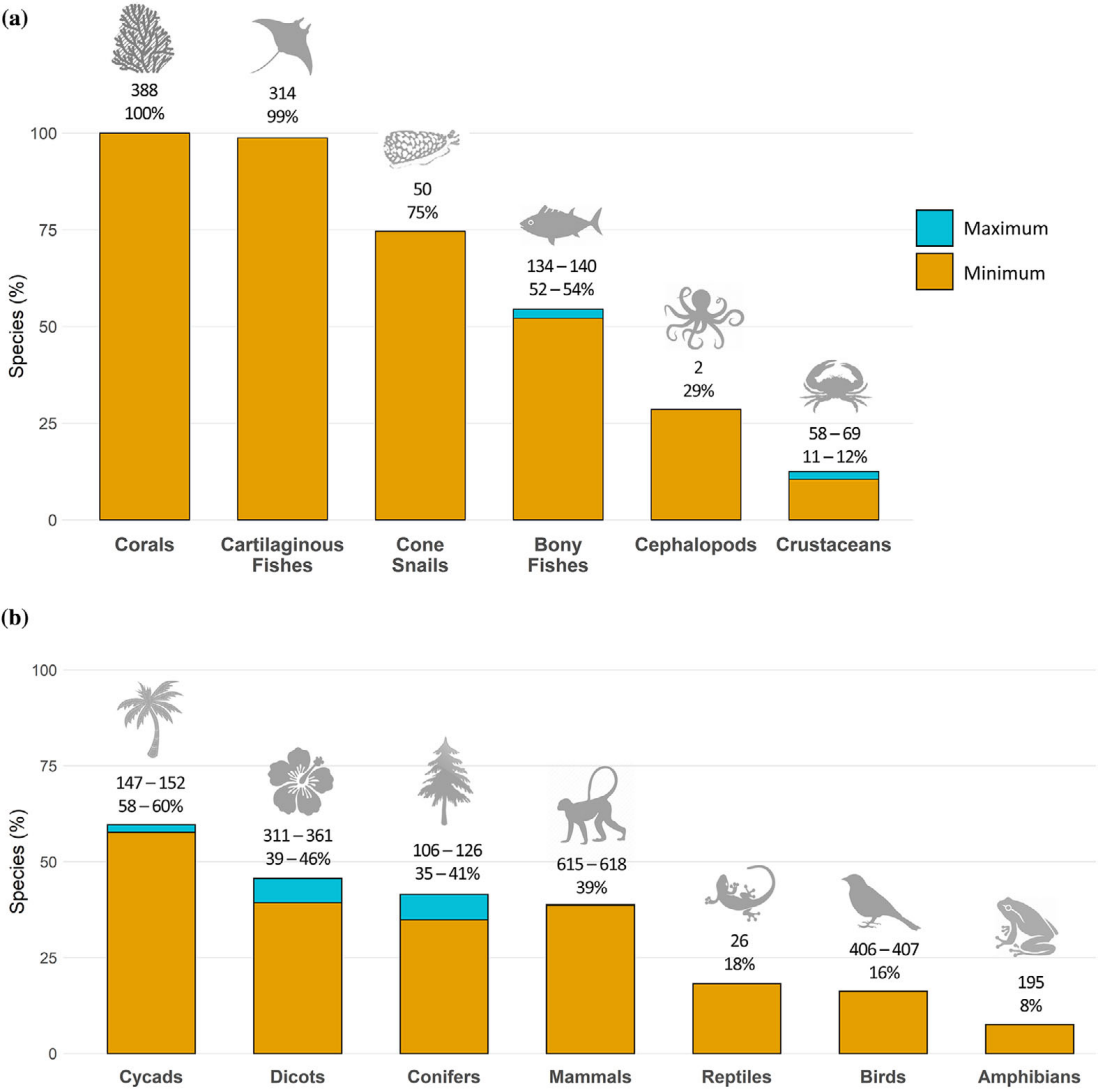
Percentage of near threatened and threatened species in (a) aquatic and (b) terrestrial groups with biological resource use documented as a threat on the International Union for Conservation of Nature Red List (orange, minimum number of species in each taxonomic group affected by at least one type of intentional use; blue, maximum number of species that might be subject to intentional use, including where species are coded as affected by use under motivation unknown; the numbers above bars, minimum number of species affected by biological resource use in each taxonomic group; bottom number, percent range from minimum to maximum [where relevant] number of species affected by biological resource use in each taxonomic group). Bony fishes, dicotyledons (dicots), and reptiles include selected higher‐level taxa (Appendix S1)

Across NT and threatened species, a higher overall proportion of aquatic species than terrestrial species had intentional BRU as a threat (Figure 2). Among aquatic groups, the taxon with highest BRU prevalence was corals (100%, 388 species). Almost all cartilaginous fishes (99%, 314 out of 318 species) had intentional BRU; harvesting was the predominant threat (Appendix S13). In the terrestrial groups, cycads appeared most affected by use (58–60%, 147–152 of 255 species), largely due to collecting (147 species).

### Wild species for which intentional use does not have a negative impact on extinction risk

**FIGURE 3:**
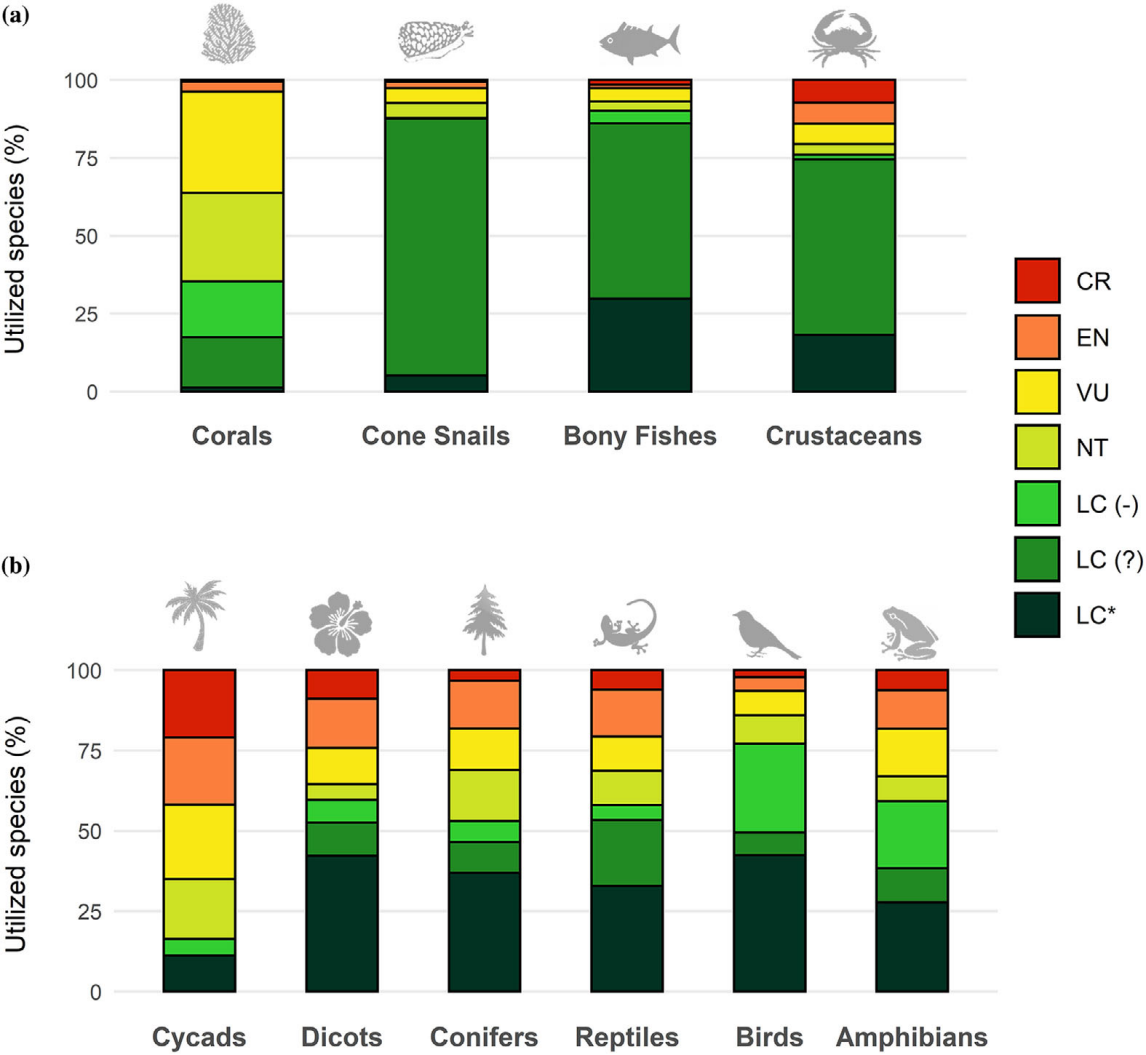
Percentage of extant, data‐sufficient species by International Union for Conservation of Nature Red List category in (a) aquatic and (b) terrestrial groups subject to use and trade (LC(‐), least concern species with declining population trend; LC(?), least concern species with unknown population trend; LC(*), least concern species with stable or increasing population trend; NT, near threatened; VU, vulnerable; EN, endangered; CR, critically endangered). Being LC and having a declining population trend or being threatened and being subject to use and trade does not imply that use is a major threat. Bony fishes, dicotyledons (dicots), and reptiles include selected higher‐level taxa (Appendix S1)

Among the 10 taxonomic groups for which information was adequate, most species subjected to some form of use or trade were LC, with the exception of cycads and corals. The percentage of utilized species that were LC was 72% (range 16% [cycads] to 76% [crustaceans]). Seventy‐seven percent of birds, 88% of cone snails, and 90% of selected bony fishes were used (Figure 3). Among terrestrial groups, from 11% (cycads, 20 species) to 42% (birds and selected dicots, 2120 and 462 species, respectively) of utilized species were LC and had either stable or increasing population trends. For aquatic groups, proportions were lower, ranging from 1% (corals, 6 species) to 30% (selected bony fishes, 413 species). Across all 10 taxa for which data on purpose of use were adequate, 34% (3469 of 10,098) of utilized species were LC and not declining. Furthermore, at least 172 threatened and NT species (2% of utilized species) were subject to some form of use but were not affected by intentional BRU and exhibited stable or increasing population trends (Table 1; list of 172 species in Appendix S9). Exclusion of assessments older than 2010 yielded proportions of species similar to the full results (χ^2^ = 37.695, df = 1, *p* < 0.0001); outdated assessments overall had higher proportions of likely unsustainable use (Appendix S14). We removed amphibians and corals from the chi‐square analysis, both of which had a higher proportion of species with outdated assessments than the red‐list average. Although removal of amphibians did not materially alter our previous results (χ^2^ = 26.202, df = 1, *p* < 0.0001), chi‐square values became insignificant when corals were excluded (χ^2^ = 2.818, df = 1, *p* = 0.093). Corals had much larger proportions of likely unsustainable use than found within our data overall (up‐to‐date assessments only: χ^2^ = 182.81, df = 1, *p* < 0.0001).

### Conservation actions in place or lacking for utilized wild species

**FIGURE 4:**
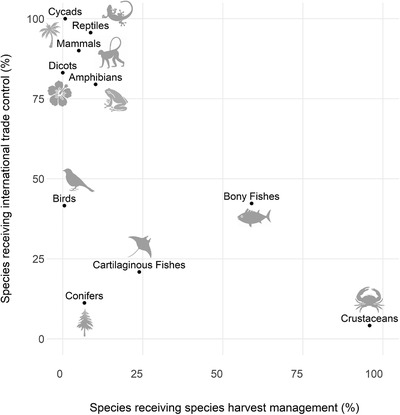
Relationship between the prevalence of international trade controls and species harvest management across near threatened and threatened species affected by intentional biological resource use (minimum estimate) based on species with available data on conservation actions (those where the field is recorded as either unknown, yes, or no, rather than left blank). No data are available for cephalopods, cone snails, or corals (Appendix S15). Bony fishes, dicotyledons (dicots), and reptiles include selected higher‐level taxa (Appendix S1)

No information on species management actions relevant to use was recorded for NT or threatened corals, cone snails, or cephalopods (Table 1). Among NT and threatened species affected by BRU, 1% of cycads (1 species), 3% of birds (12 species), and 6% of amphibians (11 species) had available information on harvest management actions (Appendix S15). There were data on international trade controls for 3% of crustaceans (2 species) and 6% of amphibians (11 species). However, over 80% of conifers, selected reptiles, and cycads and 100% of birds had documentation of species’ harvest management interventions (Appendix S15). From those species for which there were data for one or both actions, cycads, selected reptiles, mammals, and selected dicots all had >80% of species documented as being subject to some form of international trade control, and >80% of crustaceans were subject to harvest management actions (Appendix S17). Species groups were more likely to receive international trade controls (64% of species) than harvest management actions (9%), although conifers (11% and 7%, respectively), selected bony fishes (42% and 59%), and cartilaginous fishes (21% and 24%) received international trade control measures and harvest management interventions in nearly equal proportions (Figure 4).

Out of at least 2752 threatened and NT species that had intentional BRU coded as a threat (1599 of which had available documentation for one or both conservation actions), 985–989 were documented as benefitting from either international trade control or species harvest management interventions; at least 206 species were explicitly stated as lacking any such actions (Appendix S17). Compared with terrestrial groups (9% of species with available documentation), species in aquatic groups were more likely (34%) to lack management in response to BRU (Table 1).

## DISCUSSION

Although previous analyses of red‐list data mostly examined the degree to which BRU was a threatening process driving extinction risk, we provide the first attempt to use red‐list data to improve understanding of the extent to which use of wild species is not having a detrimental impact on species extinction risk. Although our analyses were hindered by data constraints, our finding that nearly three‐quarters of used species were categorized as LC indicates that, at the time of assessment, use was not placing these species at risk of extinction. Further, more than one‐third each of birds, selected reptiles, conifers, and selected dicots used were categorized as LC and exhibited either stable or increasing population trends. We also found evidence that 172 threatened and NT used species had stable or increasing population trends and did not have BRU as a major threat at the time of assessment. Red‐list assessments address species across their range; consequently, it is possible that, despite overall population trends at the time of assessment, some species could be undergoing localized declines now (or have undergone these in the past) due to the impacts of BRU (or localized increases due to successful interventions). For example, the northern chamois (*Rupicapra rupicapra*) is assessed as LC with a stable current population trend, but it is declining in parts of its range due to hunting (Anderwald et al., 2020). There could also be localized increases due to successful interventions.

In general, our results reiterate the broad extent of use of wild species. Across the assessed groups, a predominant form of use was for pets, display, or horticultural use, followed by hunting or collection for food. Among birds, the primary factor explaining the predominance of pets was the live cage‐bird trade, which has emerged as a major driver of declines among passerines, particularly in Southeast Asia (e.g., Eaton et al., 2017). Meanwhile, cacti have long been sought after for the horticultural trade and by private collectors for their ornamental value and their perceived rarity, with both seeds and mature individuals collected (Goettsch et al., 2015). Because use and trade are not consistently recorded on the Red List, especially so for nonthreatened species, we could not conclusively determine the full extent of use or the prevalence of different types of use in all comprehensively assessed taxonomic groups. However, our initial results are consistent with some taxon‐specific investigations into the use of wild species, such as trees that are often used for timber as well as horticultural purposes (e.g., Beech et al., 2017).

Although our results suggest that more species are not being affected detrimentally by use than those that are, we also found that intentional BRU was a major threat, contributing to increased extinction risk for more than one‐quarter of NT and threatened species. The proportion of species negatively affected by BRU was generally higher in aquatic taxa than among terrestrial taxa. Although the impact of fisheries is well established for bony and cartilaginous fishes (Dulvy et al., 2014; MacNeil et al., 2020), the high proportion of corals and cone snails adversely affected by BRU can generally be explained by increasing removal and harvest of corals for display in aquariums and for the curio trade in the former (Bruckner, 2000; Cannas et al., 2019) and by bioprospecting for conotoxin research and shell collecting in the latter (Peters et al., 2013).

Perhaps our starkest result was that many species that were affected by BRU had no management actions to directly address this threat. The relatively high proportion of species subject to international trade controls can be explained by the fact that the most common management action documented was listing in a CITES Appendix. All cycads, for example, are included on CITES Appendix II through a higher‐taxon listing (representing 229 out of the 255 threatened or NT cycad species in our analyses). Very few species had a national harvest management plan in place, although these appeared to be more readily available for aquatic species, such as cartilaginous fishes, which have traditionally been underrepresented in CITES. The large numbers of species negatively affected by use stresses the value of national management plans. Of course, many species affected by BRU may benefit from conservation actions that we did not directly investigate, such as establishment and management of protected areas, community‐based resource management, and other site‐based interventions, whereas some are subject to measures to reduce demand.

There are several important caveats to our analyses. First, we focused on direct and intentional forms of BRU, but the impacts of use extend well beyond impacts on the target species. The most evident examples of this are deforestation (specifically logging) in the terrestrial realm and bycatch in the aquatic realm. Although logging is clearly a major direct threat to timber species, it can also have severe repercussions on forest‐dependent species. For example, some 55% of NT and threatened bird species are affected by the unintentional effects of logging (IUCN, 2020). Likewise, while commercial fishing is a direct threat to many target fisheries, bycatch is a major recognized threatening process in the sea (Komoroske & Lewison, 2015). Our analyses included only bycatch for cartilaginous fishes where parts (e.g., fins and gills) of bycaught species frequently enter trade.

Second, we focused on 13 taxonomic groups that had been comprehensively assessed on the Red List, but our estimates of the extent of use of selected reptiles and dicotyledonous plants may be inflated because the families and orders we included were not necessarily representative of the broader diversity in the class (and were possibly more likely to be used). We also excluded DD, EX, and EW species. Although many DD species may prove to be threatened, some have been shown to be more widely distributed or common than previously understood (Butchart & Bird, 2010). Unsustainable exploitation is already understood as a driver of extinction. At least 12% (102) of species listed as recently extinct (since 1500) on the Red List have intentional BRU indicated as a threat that led to the species’ extinction (IUCN, 2020).

Third, we used information captured under the IUCN classification schemes. Because some information is mandatory and some is recommended, our analyses were constrained by the degree to which this information had been recorded consistently. Even where the information has been recorded, assessors may not always be aware of the full range of threats, uses, or actions that apply. For example, in completing the use and trade classification scheme, full consideration may not always be given to traditional or Indigenous uses. The IUCN is currently preparing guidelines that would help assessors take such uses into account. Finally, 13% of the assessments we included were over 10 years out of date; hence, the extinction risk assessments and supporting information for them may no longer be current. Although statistically the inclusion of these older assessments altered the general patterns of our findings, this was driven primarily by the patterns found for corals, a group with high levels of use and threat, most of which had not been reassessed since 2008 (Carpenter et al., 2008).

In light of our caveats, we devised a few recommendations that could help improve the utility of red‐list data for future analyses (Table 2). These recommendations focused on a small change to the threats classification scheme under class 5 to indicate where the motivation for use is known, but the scale is not (Appendix S18); making the coding of scope and severity of threats in the threats classification scheme recommended documentation for all threatened or NT species; making documentation of threats recommended information for LC species; ensuring that all assessments prioritized in the IUCN Red List Strategic Plan comply with recommended documentation requirements (which would have the benefit of also ensuring that conservation actions in place and needed are documented for all species); and adding a checkbox to indicate whether or not the classification schemes for a given species assessment have been filled in at the recommended level, which would allow for rapid determination of the completeness of the assessment supporting documentation for the purposes of analyses. We made these recommendations recognizing the time and resource constraints in the red‐list process, which is driven largely by volunteer scientists. There is a well‐documented tension between the desire to expand the taxonomic breadth of assessed species with the need to undertake timely reassessments, all the while ensuring the supporting information is as complete as possible (Rondinini et al., 2013). Given this, we cannot, for example, propose that a suite of information fields be made mandatory for assessors while acknowledging that many species urgently need reassessment. Further discussion on our recommendations is in Appendix S19.

**TABLE 2:** Recommendations for improving consistency and available information in use‐related International Union for Conservation of Nature (IUCN) Red List data^a^

Recommendation	Proposal	IUCN protocol or system affected
1	Modify threats classification scheme for classes 5.3 and 5.4 for assessors to indicate where the motivation is known, but the scale is not.	threats classification scheme (class 5)
2	Coding of scope and severity of threats becomes recommended information for taxa listed as EX, EW, threatened, or NT.	required and recommended supporting information for IUCN Red List assessments (annex 1 of rules of procedure for IUCN Red List assessments)
3	Documentation of threats (with timing, scope, and severity) becomes recommended information for LC species.	required and recommended supporting information for IUCN Red List assessments (annex 1 of rules of procedure for IUCN Red List assessments)
4	Assessments for taxa prioritized in the IUCN Red List Strategic Plan comply with the recommended documentation requirements.	no change (support for current protocol)
5	Addition of a checkbox to the species information service to indicate whether or not the classification schemes for a given species assessment have been filled in at the recommended level.	species information service

^a^ The IUCN Red List's categories of species extinction risk: LC, least concern; NT, near threatened; EX, extinct; EW, extinct in the wild.

As previous studies have shown, red‐list data can play a key role in supporting major global assessment processes and by extension broader international policy. We used red‐list data to quantify the degree to which the use of wild species either did or did not negatively affect species extinction risk and thus whether documented use may be biologically sustainable or unsustainable. The ability to disentangle the nature and extent of this use could be considerably improved through minor amendments to established red‐list protocols and through greater efforts by assessors and red‐list assessment initiatives to ensure, wherever possible, more consistent recording of the information and data underpinning assessments. Nonetheless, our findings show that while overexploitation is clearly a direct major threat to many species and has already driven some to extinction, there are also many species for which at the time of assessment use was taking place at levels that were unlikely to contribute significantly to an increase in their extinction risk.

Our results can usefully inform processes, such as the IPBES assessment on sustainable use, and our analyses provide important information for international policy making because the Red List is frequently taken into consideration in policy deliberations, such as the progress of species through the CITES Review of Significant Trade process (CITES, 2018). However, there is no reason these data could not also be harvested to inform other decision‐making, both within but also beyond CITES. We provide a first attempt at disentangling likely sustainable versus likely unsustainable use of species based on information on IUCN's Red List. Ideally, this information should be combined with other data sources, including national or regional data sets, to provide the most complete picture of the impacts of use of wild species possible.

## Supporting information

Supplementary material

Supplementary material
