# Supplementary material for: Prevalence of sustainable and unsustainable use of wild species inferred from the IUCN Red List of Threatened Species
Source: Conserv Biol. 2021 Nov 17;36(2):e13844. doi: 10.1111/cobi.13844 (PMC9299080; doi:10.1111/cobi.13844)
Supplement: Supplementary file 1 — Supplementary material [file COBI-36-0-s001.docx]

**Supporting information**

**Appendix S1.** Taxonomic description of species in comprehensively assessed groups of the International Union for Conservation of Nature (IUCN) Red List of Threatened Species.

| Taxonomic group | Phylum | Class | Order | Family | Genus |
| --- | --- | --- | --- | --- | --- |
| Cephalopods | Mollusca | Cephalopoda* | All species | | |
| Cone snails | Mollusca | Gastropoda | Neogastropoda | Conidae | Conus |
| Corals | Cnidaria | Hydrozoa | All species | | |
|  |  | Anthozoa | Helioporacea | All species | |
|  |  |  | Scleractinia | Acroporidae | All species |
|  |  |  |  | Agariciidae | All species |
|  |  |  |  | Astrocoeniidae | All species |
|  |  |  |  | Euphyllidae | All species |
|  |  |  |  | Faviidae | All species |
|  |  |  |  | Mussidae | All species |
|  |  |  |  | Oculinidae | All species |
|  |  |  |  | Pectiniidae | All species |
|  |  |  |  | Pocilloporidae | All species |
|  |  |  |  | Poritidae | All species |
|  |  |  |  | Rhizangiidae | All species |
|  |  |  |  | Siderastreidae | All species |
|  |  |  |  | Trachyphylliidae | All species |
|  |  |  |  | Turbinoliidae | All species |
|  |  |  |  | Dendrophylliidae | Balanophyllia |
|  |  |  |  |  | Duncanopsammia |
|  |  |  |  |  | Heteropsammia |
|  |  |  |  |  | Turbinaria |
|  |  |  |  | Caryophylliidae | Heterocyathus |
| Cartilaginous fishes (sharks, rays, chimaeras) | Chordata | Chondrichthyes | All species | | |
| Bony fishes (selected): |  |  |  |  |  |
| Sturgeons | Chordata | Actinopterygii | Acipenseriformes | All species | |
| Tarpons & bonefishes |  |  | Albuliformes | All species | |
|  |  |  | Elopiformes | All species | |
| Anchovies, sardines etc. |  |  | Clupeiformes | All species | |
| Groupers & wrasses |  |  | Perciformes | Epinephelidae | All species |
|  |  |  |  | Labridae | All species |
| Tunas |  |  |  | Scombridae | All species |
| Billfishes |  |  |  | Istiophoridae | All species |
|  |  |  |  | Xiphiidae | All species |
| Blennies |  |  |  | Blenniidae | All species |
|  |  |  |  | Chaenopsidae | All species |
|  |  |  |  | Clinidae | All species |
|  |  |  |  | Dactyloscopidae | All species |
|  |  |  |  | Labrisomidae | All species |
|  |  |  |  | Tripterygiidae | All species |
| Seabreams |  |  |  | Sparidae | All species |
|  |  |  |  | Centracanthidae | All species |
| Angelfishes |  |  |  | Pomacanthidae | All species |
| Butterflyfishes |  |  |  | Chaetodontidae | All species |
| Surgeonfishes |  |  |  | Acanthuridae | All species |
| Pufferfishes |  |  | Tetraodontiformes | Tetraodontidae | All species |
| Seahorses & pipefish |  |  | Syngnathiformes | Syngnathidae | All species |
| Trumpetfishes |  |  |  | Aulostomidae | All species |
| Shrimpfishes |  |  |  | Centriscidae | All species |
| Seamoths |  |  |  | Pegasidae | All species |
| Ghost pipefishes |  |  |  | Solenostomidae | All species |
| Cornetfishes |  |  | Gasterosteiformes | Fistulariidae | All species |
| Crustaceans (selected): |  |  |  |  |  |
| Lobsters | Arthropoda | Malacostraca | Decapoda | Glypheidae | All species |
|  |  |  |  | Polychelidae | All species |
|  |  |  |  | Nephropidae | All species |
|  |  |  |  | Enoplometopidae | All species |
|  |  |  |  | Palinuridae | All species |
|  |  |  |  | Scyllaridae | All species |
| Freshwater crayfishes |  |  |  | Astacidae | All species |
|  |  |  |  | Cambaridae | All species |
|  |  |  |  | Parastacidae | All species |
| Freshwater crabs |  |  |  | Trichodactylidae | All species |
|  |  |  |  | Potamidae | All species |
|  |  |  |  | Potamonautidae | All species |
|  |  |  |  | Gecarcinucidae | All species |
|  |  |  |  | Pseudothelphusidae | All species |
| Freshwater shrimps |  |  |  | Alpheidae | FW species only |
|  |  |  |  | Atyidae | FW species only |
|  |  |  |  | Desmocarididae | FW species only |
|  |  |  |  | Euryrhynchidae | FW species only |
|  |  |  |  | Palaemonidae | FW species only |
|  |  |  |  | Typhlocarididae | FW species only |
|  |  |  |  | Xiphocarididae | FW species only |
| Amphibians | Chordata | Amphibia | All species | | |
| Birds | Chordata | Aves | All species | | |
| Mammals | Chordata | Mammalia | All species | | |
| Conifers | Tracheophyta | Pinopsida | All species | | |
| Cycads |  | Cycadopsida | All species | | |
| Dicotyledons (selected): |  |  |  |  |  |
| Cacti | Tracheophyta | Magnoliopsida | Caryophyllales | Cactaceae | All species |
| Magnolias |  |  | Magnoliales | Magnoliaceae | All species |
| Birches |  |  | Fagales | Betulaceae | All species |
| Southern beeches |  |  |  | Nothofagaceae | All species |
| Teas |  |  | Ericales | Theaceae | All species |
| Reptiles (selected): |  |  |  |  |  |
| Chameleons | Chordata | Reptilia | Squamata | Chamaeleonidae | All species |
| Sea snakes |  |  |  | Homalopsidae | All species |
|  |  |  |  | Elapidae | Aipysurus |
|  |  |  |  |  | Emydocephalus |
|  |  |  |  |  | Ephalophis |
|  |  |  |  |  | Hydrelaps |
|  |  |  |  |  | Hydrophis |
|  |  |  |  |  | Kerilia |
|  |  |  |  |  | Kolpophis |
|  |  |  |  |  | Laticauda |
|  |  |  |  |  | Parahydrophis |
|  |  |  |  |  | Thalassophis |
|  |  |  |  | Acrochordidae | Acrochordus |
|  |  |  |  | Natricidae | Anoplohydrus |
| Crocodiles & alligators |  |  | Crocodylia | All species | |
| Marine turtles |  |  | Testudines | Cheloniidae | All species |
|  |  |  |  | Dermochelyidae | All species |

*Nautiluses are not yet assessed

**Appendix S2.** The International Union for Conservation of Nature (IUCN)’s threats classification scheme class 5 following Salafsky et al. (2008)

| Threats classification scheme 5. Biological resource use | |
| --- | --- |
| 5.1 | Hunting & collecting terrestrial animals  5.1.1 Intentional use (species being assessed is the target)  5.1.2 Unintentional effects (species being assessed is not the target)  5.1.3 Persecution and control  5.1.4 Motivation unknown or unrecorded |
| 5.2 | Gathering terrestrial plants  5.2.1 Intentional use (species being assessed is the target)  5.2.2 Unintentional effects (species being assessed is not the target)  5.2.3 Persecution and control  5.2.4 Motivation unknown or unrecorded |
| 5.3 | Logging & wood harvesting  5.3.1 Intentional use: subsistence and small scale (species being assessed is the target)  5.3.2 Intentional use: large scale (species being assessed is the target)  5.3.3 Unintentional effects: subsistence and small scale (species being assessed is not the target)  5.3.4 Unintentional effects: large scale (species being assessed is not the target)  5.3.5 Motivation unknown or unrecorded |
| 5.4 | Fishing & harvesting aquatic resources  5.4.1 Intentional use: subsistence and small scale (species being assessed is the target)  5.4.2 Intentional use: large scale (species being assessed is the target)  5.4.3 Unintentional effects: subsistence and small scale (species being assessed is not the target)  5.4.4 Unintentional effects: large scale (species being assessed is not the target)  5.4.5 Persecution and control  5.4.6 Motivation unknown or unrecorded |

**Appendix S3.** The International Union for Conservation of Nature (IUCN)’s use and trade classification scheme. Analyses exclude use codes 16 through 18.

| 1. Food – human 2. Food – animal 3. Medicine – human & veterinary 4. Poisons 5. Manufacturing chemicals 6. Other chemicals 7. Fuels 8. Fibre 9. Construction or structural materials | 1. Wearing apparel, accessories 2. Other household goods 3. Handicrafts, jewellery, etc. 4. Pets or display animals, horticulture 5. Research 6. Sport hunting and specimen collecting 7. Establishing ex situ production 8. Other (free text) 9. Unknown |
| --- | --- |

**Appendix S4.** Supplementary methods: the main purposes of use of wild animal and plant species recorded in the International Union for Conservation of Nature (IUCN) Red List of Threatened Species

We investigated the prevalence of different purposes of use from the use and trade classification scheme recorded in the Red List of Threatened Species (Red List). Because completing the use and trade classification scheme is not mandatory for International Union for Conservation of Nature (IUCN) assessors, we investigated the prevalence of use and trade recording to decide which species groups to include in our analyses. We selected taxonomic groups for inclusion based on the following criteria: >40% of all extant, Data-sufficient species have at least one purpose of use coded (thus selecting taxonomic groups with high prevalence of use); or the proportion of least concern (LC) species with at least one purpose of use falls above or within the range of the proportion of species with use and trade documentation across the other Red List categories (thus also selecting taxonomic groups where use and trade may be relatively low, but use and trade in LC species is coded to a similar level as that of species in other Red List categories). This limited our dataset to the following taxonomic groups which have adequate recording of use and trade: birds, amphibians, selected reptiles, cycads, conifers and selected dicots from the terrestrial group; and corals, selected bony fishes, crustaceans and cone snails from the aquatic species group (Appendix S5). We excluded mammals, cephalopods and cartilaginous fishes as they meet neither criterion, meaning within these groups there is either relatively little use, or Red List documentation of use is incomplete.

For each taxonomic group, we calculated the total number of species recorded as being used for at least one purpose in the use and trade classification scheme. However, we excluded use for the purpose of establishing ex-situ production (use code 16 [Appendix S3]), other (17), and unknown (18). Ex-situ production involves establishing captive populations for conservation breeding and translocation but also for ranching, farming or propagation purposes. It is not possible to distinguish these purposes without the IUCN assessors voluntarily providing information on harvest from captive and cultivated sources. Only eleven species (six cartilaginous fishes, four bony fishes, and one bird) in our analyses had an unknown use and trade category. We summarized the data as the percentage of species recorded for different types of use on the Red List.

**Appendix S5.** Proportion of extant, Data-sufficient species in each taxonomic group with at least one purpose of use documented on the International Union for Conservation of Nature (IUCN)’s use and trade classification scheme. ^^[[1]](#footnote-1)^^

| **Taxonomic group** | **Proportion LC** | **Proportion threatened** | **Proportion all species** | **LC in range** | **Criterion i** | **Criterion ii** |
| --- | --- | --- | --- | --- | --- | --- |
| Amphibians | 0.12 | 0.09 | 0.11 | Within | No | Yes |
| Birds | 0.46 | 0.47 | 0.46 | Within | Yes | Yes |
| Selected bony fishes | 0.53 | 0.48 | 0.53 | Within | Yes | Yes |
| Cartilaginous fishes | 0.22 | 0.51 | 0.35 | Below | No | No |
| Cephalopods | 0.20 | 0.20 | 0.21 | Below | No | No |
| Cone snails | 1.00 | 0.98 | 1.00 | Within | Yes | Yes |
| Conifers | 0.82 | 0.69 | 0.76 | Above | Yes | Yes |
| Corals | 0.67 | 0.79 | 0.75 | Within | Yes | Yes |
| Crustaceans | 0.17 | 0.11 | 0.15 | Above | No | Yes |
| Cycads | 0.69 | 0.85 | 0.80 | Below | Yes | No |
| Selected dicots | 0.59 | 0.58 | 0.58 | Within | Yes | Yes |
| Mammals | 0.21 | 0.43 | 0.28 | Below | No | No |
| Selected reptiles | 0.53 | 0.41 | 0.46 | Above | Yes | Yes |


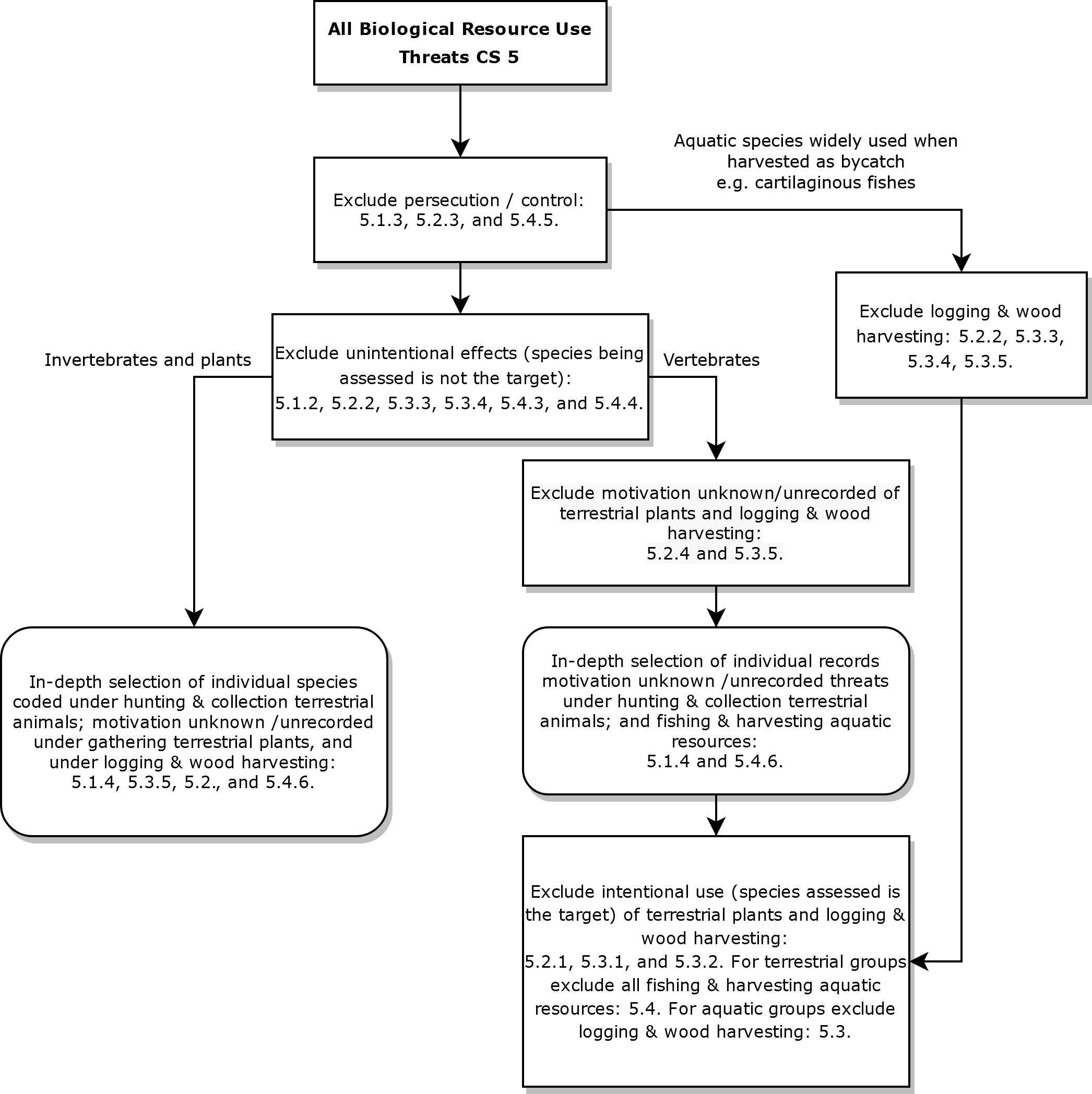


**Appendix S6.** Flow chart describing the process for including forms of biological resource use (based on the International Union for Conservation of Nature’s threats classification scheme 5) that directly target the species in the taxonomic groups selected, focusing on intentional forms of use. In-depth selection entails checking a species’ threat categories against detailed text information in the assessment records.

**Appendix S7.** Supplementary methods: wild species for which intentional use is having a negative impact on extinction risk

Because not all types of biological resource use (BRU) directly target species, we developed a decision-tree (Appendix S6) to remove threat types that are not relevant to analyses of sustainable and unsustainable direct and intentional use of species.

First, we discounted the threat of persecution or control, which does not represent intentional consumptive BRU. We excluded threats where the species was not the deliberate target of activity, here termed unintentional BRU, with the exception of aquatic species groups which are widely used even if unintentionally caught as bycatch (e.g., cartilaginous fishes; Dulvy et al. 2014). We also excluded records of vertebrates threatened by intentional gathering of plants, or by logging and wood harvesting, as these likely represent assessment errors and should have been coded as unintentional.

In addition to intentional and unintentional use, the IUCN’s threats classification scheme also allows for BRU to be recorded if the motivation behind use is unknown (i.e., the IUCN’s assessors do not know if use is intentionally targeting a species). While intentional gathering of terrestrial plants or logging and wood harvesting (threat codes 5.2.4 and 5.3.5) can threaten animals through habitat loss, they do not directly use animals and we excluded those records for animal groups. Where BRU was recorded with unknown motivation (codes 5.1.4 and 5.4.6 for animal species; codes 5.2.4 and 5.3.5 for plant species [Appendix S2]), we reviewed the detailed text of individual species’ assessments to determine whether these records should be excluded from the analysis. For instance, Red List documentation records the Pygmy Slow Loris (*Nycticebus pygmaeus*) as threatened by intentional hunting and collecting of terrestrial animals (5.1.1), and by fishing and harvesting of aquatic resources for which the motivation is unknown or unrecorded (5.4.6). This terrestrial species is exploited for the pet trade and harvested for medicinal use, hunted as a food source, and experiences habitat loss due to agriculture. Its categorization under 5.4.6 likely represents a threat coding error and we excluded it, as no aquatic resource use is known to directly affect this terrestrial species. For some BRU threats with unknown motivation, we could not determine whether the threat directly targeted the species. We present this uncertainty in our results as a range where the minimum proportion includes all species with threats that could be conclusively determined as intentional (and hence more evidentiary), and the maximum proportion additionally includes species with use motivation unknown or unrecorded that may represent further cases of intentional BRU (and hence more precautionary). For groups that had no species with such threats, we present only the minimum.

We only included BRU if it had a major impact on species survival. The Red List uses a scoring system to derive threat impact, based on three elements: threat timing, categorized as past (unlikely to return), past (likely to return), ongoing, future, or unknown; threat scope, categorized as the percentage of the population affected by the threat, i.e., whole, >90%, majority, 50-90%, minority, <50%, or unknown; and threat severity, categorized as causing very rapid declines, rapid declines, slow and significant declines, causing or would cause fluctuations or negligible declines, or unknown. The IUCN uses this information to produce an overall threat impact score by summing the scores for timing, scope and severity for each threat impacting a species (IUCN 2020). While threat timing is recommended information to be provided in the Red List assessment (strongly encouraged but not essential to publication), severity and scope are optional. Because of this we utilized the impact score categorization with several amendments (Appendix S8). We treated threats classed as in the past but likely to return the same as future threats, awarding them a score of 1; we assigned a medium score of 2 to threats with either unknown or missing timing, severity and scope information. Subsequently, we excluded threats with an impact score signifying low, negligible or no impact, and retained medium to high impact threats (threat impact score >= 6). As an example, the amphibian *Herpele squalostoma* was threatened by intentional use (5.4.2, where the species is the target): this was an ongoing threat (timing) associated with a minority scope (<50% of population affected), causing negligible declines (severity). We calculated an impact score of 4 and excluded this threat from analysis. The Northern Fur Seal *Callorhinus ursinus* was threatened by large-scale intentional BRU (5.4.2) in the past (unlikely to return); a threat of majority scope, possibly causing fluctuations. This threat also yielded an impact score of 4 and was excluded. The main implication of our amended scoring is that it was precautionary in including threats where both severity and scope were marked as unknown (qualified as medium impact), but evidentiary in excluding threats if at least one of them was coded as negligible or minority.

**Appendix S8.** Scoring system for the impact of threats used in the current analysis, adapted from the International Union for Conservation of Nature (IUCN) Red List^^[[2]](#footnote-2)^^.

| Timing: | | Ongoing threat (+3) | | | | Future or past, likely to return threat (long term) (+1) | | | | |
| --- | --- | --- | --- | --- | --- | --- | --- | --- | --- | --- |
| Severity:  Scope: | *Very rapid*  (+3) | | *Rapid or unknown*  (+2) | *Slow or fluctuating*  (+1) | *Negligible or no impact* (0) | | *Very rapid*  (+3) | *Rapid or Unknown*  (+2) | *Slow or Fluctuating*  (+1) | *Negligible or no impact* (0) |
| Whole (+3) | 9 | | 8 | 7 | 6 | | 7 | 6 | 5 | 4 |
| Majority or unknown (+2) | 8 | | 7 | 6 | 5 | | 6 | 5 | 4 | 3 |
| Minority (+1) | 7 | | 6 | 5 | 4 | | 5 | 4 | 3 | 2 |

Additive impact scores:

8-9: High impact threat

6-7: Medium impact threat

3-5: Low impact threat

0-2: Negligible or no impact threat

**Appendix S9.** Threatened or near threatened species with at least one purpose of use or trade recorded that are not impacted by major, intentional biological resource use, and have stable or increasing population trends at the time of assessment.

| **Taxonomic group** | **Scientific name** | **Red List Category** | **Population trend** |
| --- | --- | --- | --- |
| Amphibians | *Leptobrachium ailaonicum* | Near Threatened | Stable |
| Amphibians | *Atelopus flavescens* | Vulnerable | Stable |
| Amphibians | *Eurycea rathbuni* | Vulnerable | Stable |
| Amphibians | *Lyciasalamandra helverseni* | Vulnerable | Stable |
| Amphibians | *Lyciasalamandra luschani* | Vulnerable | Stable |
| Amphibians | *Lyciasalamandra atifi* | Endangered | Stable |
| Birds | *Accipiter collaris* | Near Threatened | Stable |
| Birds | *Accipiter poliogaster* | Near Threatened | Increasing |
| Birds | *Acrocephalus rodericanus* | Near Threatened | Increasing |
| Birds | *Actinodura sodangorum* | Near Threatened | Stable |
| Birds | *Anas chlorotis* | Near Threatened | Increasing |
| Birds | *Buteo solitarius* | Near Threatened | Stable |
| Birds | *Callaeas wilsoni* | Near Threatened | Increasing |
| Birds | *Caprimulgus phalaena* | Near Threatened | Stable |
| Birds | *Charadrius aquilonius* | Near Threatened | Increasing |
| Birds | *Charadrius melodus* | Near Threatened | Increasing |
| Birds | *Charadrius pallidus* | Near Threatened | Stable |
| Birds | *Chloebia gouldiae* | Near Threatened | Stable |
| Birds | *Cynanthus lawrencei* | Near Threatened | Stable |
| Birds | *Dendrocopos owstoni* | Near Threatened | Stable |
| Birds | *Dendropicos stierlingi* | Near Threatened | Stable |
| Birds | *Ducula whartoni* | Near Threatened | Stable |
| Birds | *Egretta rufescens* | Near Threatened | Increasing |
| Birds | *Elanus scriptus* | Near Threatened | Stable |
| Birds | *Erythrura coloria* | Near Threatened | Stable |
| Birds | *Estrilda poliopareia* | Near Threatened | Stable |
| Birds | *Eudyptes schlegeli* | Near Threatened | Stable |
| Birds | *Euplectes jacksoni* | Near Threatened | Stable |
| Birds | *Foudia flavicans* | Near Threatened | Increasing |
| Birds | *Fringilla teydea* | Near Threatened | Increasing |
| Birds | *Garrulax nuchalis* | Near Threatened | Stable |
| Birds | *Gyps himalayensis* | Near Threatened | Stable |
| Birds | *Hemiphaga novaeseelandiae* | Near Threatened | Increasing |
| Birds | *Hypsipetes borbonicus* | Near Threatened | Increasing |
| Birds | *Larus atlanticus* | Near Threatened | Stable |
| Birds | *Larvivora komadori* | Near Threatened | Stable |
| Birds | *Megascops marshalli* | Near Threatened | Stable |
| Birds | *Microcarbo coronatus* | Near Threatened | Stable |
| Birds | *Oxyura australis* | Near Threatened | Stable |
| Birds | *Parotia wahnesi* | Near Threatened | Stable |
| Birds | *Phalcoboenus australis* | Near Threatened | Stable |
| Birds | *Philesturnus carunculatus* | Near Threatened | Increasing |
| Birds | *Philesturnus rufusater* | Near Threatened | Increasing |
| Birds | *Phoebastria immutabilis* | Near Threatened | Stable |
| Birds | *Phoebastria nigripes* | Near Threatened | Increasing |
| Birds | *Polytelis alexandrae* | Near Threatened | Stable |
| Birds | *Psittinus abbotti* | Near Threatened | Stable |
| Birds | *Pyrilia caica* | Near Threatened | Stable |
| Birds | *Pyrrhura devillei* | Near Threatened | Stable |
| Birds | *Speculanas specularis* | Near Threatened | Stable |
| Birds | *Streptopelia reichenowi* | Near Threatened | Stable |
| Birds | *Thalasseus elegans* | Near Threatened | Stable |
| Birds | *Todiramphus pelewensis* | Near Threatened | Stable |
| Birds | *Amazona arausiaca* | Vulnerable | Increasing |
| Birds | *Amazona guildingii* | Vulnerable | Increasing |
| Birds | *Anas albogularis* | Vulnerable | Stable |
| Birds | *Anas aucklandica* | Vulnerable | Stable |
| Birds | *Anthropoides paradiseus* | Vulnerable | Stable |
| Birds | *Ardenna bulleri* | Vulnerable | Stable |
| Birds | *Branta sandvicensis* | Vulnerable | Increasing |
| Birds | *Buteo galapagoensis* | Vulnerable | Stable |
| Birds | *Capito wallacei* | Vulnerable | Stable |
| Birds | *Charadrius sanctaehelenae* | Vulnerable | Increasing |
| Birds | *Coracopsis barklyi* | Vulnerable | Stable |
| Birds | *Cyanoramphus unicolor* | Vulnerable | Stable |
| Birds | *Diomedea epomophora* | Vulnerable | Stable |
| Birds | *Eunymphicus cornutus* | Vulnerable | Increasing |
| Birds | *Eunymphicus uvaeensis* | Vulnerable | Increasing |
| Birds | *Falco araeus* | Vulnerable | Stable |
| Birds | *Falco hypoleucos* | Vulnerable | Stable |
| Birds | *Fregata aquila* | Vulnerable | Stable |
| Birds | *Fulica alai* | Vulnerable | Stable |
| Birds | *Grus monacha* | Vulnerable | Increasing |
| Birds | *Hemiphaga chathamensis* | Vulnerable | Increasing |
| Birds | *Icterus oberi* | Vulnerable | Stable |
| Birds | *Leucocarbo campbelli* | Vulnerable | Stable |
| Birds | *Leucocarbo colensoi* | Vulnerable | Stable |
| Birds | *Leucocarbo ranfurlyi* | Vulnerable | Stable |
| Birds | *Lonchura vana* | Vulnerable | Stable |
| Birds | *Megapodius pritchardii* | Vulnerable | Increasing |
| Birds | *Nannopterum harrisi* | Vulnerable | Stable |
| Birds | *Nesoenas mayeri* | Vulnerable | Stable |
| Birds | *Ninox natalis* | Vulnerable | Stable |
| Birds | *Odontophorus dialeucos* | Vulnerable | Stable |
| Birds | *Phoebastria albatrus* | Vulnerable | Increasing |
| Birds | *Phoenicoparrus andinus* | Vulnerable | Stable |
| Birds | *Pica nutalli* | Vulnerable | Stable |
| Birds | *Poicephalus robustus* | Vulnerable | Stable |
| Birds | *Procellaria parkinsoni* | Vulnerable | Stable |
| Birds | *Psittacula eques* | Vulnerable | Increasing |
| Birds | *Pterodroma axillaris* | Vulnerable | Increasing |
| Birds | *Pterodroma deserta* | Vulnerable | Stable |
| Birds | *Pterodroma solandri* | Vulnerable | Increasing |
| Birds | *Pyrrhula murina* | Vulnerable | Stable |
| Birds | *Pyrrhura perlata* | Vulnerable | Stable |
| Birds | *Scolopax mira* | Vulnerable | Stable |
| Birds | *Thalassarche eremita* | Vulnerable | Stable |
| Birds | *Touit huetii* | Vulnerable | Stable |
| Birds | *Alopecoenas rubescens* | Endangered | Stable |
| Birds | *Copsychus sechellarum* | Endangered | Increasing |
| Birds | *Cyclopsitta coxeni* | Endangered | Stable |
| Birds | *Foudia aldabrana* | Endangered | Increasing |
| Birds | *Fringilla polatzeki* | Endangered | Increasing |
| Birds | *Grus americana* | Endangered | Increasing |
| Birds | *Hypotaenidia sylvestris* | Endangered | Stable |
| Birds | *Junco insularis* | Endangered | Increasing |
| Birds | *Nipponia nippon* | Endangered | Increasing |
| Birds | *Ognorhynchus icterotis* | Endangered | Increasing |
| Birds | *Papasula abbotti* | Endangered | Stable |
| Birds | *Platalea minor* | Endangered | Increasing |
| Birds | *Porphyrio hochstetteri* | Endangered | Stable |
| Birds | *Pterodroma cahow* | Endangered | Increasing |
| Birds | *Pterodroma madeira* | Endangered | Stable |
| Birds | *Rhyticeros narcondami* | Endangered | Stable |
| Birds | *Xenoperdix udzungwensis* | Endangered | Stable |
| Birds | *Amazilia alfaroana* | Critically Endangered | Stable |
| Birds | *Aythya innotata* | Critically Endangered | Stable |
| Birds | *Cyanoramphus malherbi* | Critically Endangered | Stable |
| Birds | *Gymnogyps californianus* | Critically Endangered | Increasing |
| Birds | *Hypotaenidia owstoni* | Critically Endangered | Increasing |
| Birds | *Pterodroma magentae* | Critically Endangered | Increasing |
| Birds | *Strigops habroptila* | Critically Endangered | Increasing |
| Birds | *Vini ultramarina* | Critically Endangered | Stable |
| Bony Fishes | *Centropyge nahackyi* | Near Threatened | Stable |
| Bony Fishes | *Holacanthus limbaughi* | Near Threatened | Stable |
| Bony Fishes | *Holacanthus clarionensis* | Vulnerable | Stable |
| Cone Snails | *Conus kersteni* | Near Threatened | Stable |
| Cone Snails | *Conus fontonae* | Vulnerable | Stable |
| Cone Snails | *Conus teodorae* | Vulnerable | Stable |
| Cone Snails | *Conus xicoi* | Vulnerable | Stable |
| Conifers | *Abies bracteata* | Near Threatened | Stable |
| Conifers | *Abies kawakamii* | Near Threatened | Stable |
| Conifers | *Agathis atropurpurea* | Near Threatened | Stable |
| Conifers | *Agathis microstachya* | Near Threatened | Stable |
| Conifers | *Austrocedrus chilensis* | Near Threatened | Increasing |
| Conifers | *Chamaecyparis lawsoniana* | Near Threatened | Increasing |
| Conifers | *Cryptomeria japonica* | Near Threatened | Stable |
| Conifers | *Halocarpus kirkii* | Near Threatened | Stable |
| Conifers | *Libocedrus bidwillii* | Near Threatened | Stable |
| Conifers | *Libocedrus plumosa* | Near Threatened | Increasing |
| Conifers | *Pherosphaera hookeriana* | Near Threatened | Stable |
| Conifers | *Pinus balfouriana* | Near Threatened | Stable |
| Conifers | *Pinus jaliscana* | Near Threatened | Stable |
| Conifers | *Amentotaxus formosana* | Vulnerable | Stable |
| Conifers | *Callitris oblonga* | Vulnerable | Stable |
| Conifers | *Cupressus macrocarpa* | Vulnerable | Stable |
| Conifers | *Prumnopitys ladei* | Vulnerable | Stable |
| Conifers | *Thuja sutchuenensis* | Endangered | Increasing |
| Conifers | *Juniperus bermudiana* | Critically Endangered | Increasing |
| Cycads | *Encephalartos septentrionalis* | Near Threatened | Stable |
| Cycads | *Macrozamia longispina* | Near Threatened | Stable |
| Cycads | *Zamia pseudomonticola* | Near Threatened | Stable |
| Cycads | *Cycas semota* | Vulnerable | Stable |
| Dicots | *Cleistocactus acanthurus* | Near Threatened | Stable |
| Dicots | *Echinocactus parryi* | Near Threatened | Stable |
| Dicots | *Echinocereus websterianus* | Near Threatened | Stable |
| Dicots | *Mammillaria boolii* | Near Threatened | Stable |
| Dicots | *Pachycereus lepidanthus* | Near Threatened | Stable |
| Dicots | *Parodia columnaris* | Near Threatened | Stable |
| Dicots | *Rebutia arenacea* | Near Threatened | Stable |
| Dicots | *Rhipsalis olivifera* | Near Threatened | Stable |
| Dicots | *Cephalocereus nizandensis* | Vulnerable | Stable |
| Dicots | *Discocactus horstii* | Vulnerable | Stable |
| Dicots | *Gymnocalycium marianae* | Vulnerable | Stable |
| Dicots | *Mammillaria multidigitata* | Vulnerable | Stable |
| Dicots | *Mammillaria tayloriorum* | Vulnerable | Stable |
| Dicots | *Neobuxbaumia polylopha* | Vulnerable | Stable |
| Dicots | *Schlumbergera microsphaerica* | Vulnerable | Stable |
| Dicots | *Cleistocactus sulcifer* | Endangered | Stable |
| Reptiles | *Bradypodion dracomontanum* | Near Threatened | Stable |
| Reptiles | *Furcifer timoni* | Near Threatened | Stable |
| Reptiles | *Kinyongia oxyrhina* | Near Threatened | Stable |

**Appendix S10.** Summary of analyses undertaken

We provide a short-hand summary of the main analyses undertaken in this paper, which correspond to the numbers at the bottom of Table 1.

1. The main purposes of use of wild animal and plant species on the Red List

Estimated as: the total number of species recorded as being used for at least one purpose of use as the percentage of extant, data-sufficient species recorded for different types of use (Fig. 1)

2. Wild species for which intentional use is having a negative impact on extinction risk (i.e., for which BRU is documented as a major threat), estimated from among:

1. all extant, data-sufficient species with at least one purpose of use coded (sensu analysis 1);
2. all NT and threatened species (from among all 13 taxonomic groups comprehensively assessed; Fig. 2, Appendix S13)

3. Wild species for which intentional BRU is not having a negative impact on extinction risk. Estimated from the number of extant, Data-sufficient species recorded as being subject to some form of use or trade that:

1. are currently LC;
2. currently LC and not declining (i.e., have either stable or increasing current population trends at the time of assessment; Fig. 3);
3. are threatened or NT and are not documented as having intentional BRU as a major threat and have stable or increasing population trends at the time of assessment.

4. Conservation actions in place or lacking for utilized wild species.

Estimated from the number of NT and threatened species that are adversely affected by biological resource use and:

1. are coded as receiving either one or both of international trade controls and / or harvest management actions (Fig. 4);
2. are not receiving either of these conservation management actions.

**Appendix S11.** Glossary of Red List-related terms used in the manuscript

Biological resource use: threats from consumptive use of “wild” biological resources including deliberate and unintentional harvesting effects; also, persecution or control of specific species (Salafsky et al. 2008). Part of the IUCN-Conservation Measures Partnership (CMP) classification of direct threats to biodiversity and the IUCN Red List Threat Classification Scheme (Class 5). See Appendix S2.

Comprehensively assessed / comprehensive assessment: taxonomic groups that include at least 150 species, of which >80% have been assessed (IUCN 2020).

Conservation Actions In Place Classification Scheme*:* Used by assessors to record what conservation actions are already in place for a taxon. This information is not mandatory, only recommended. For definitions, examples and guidance on the Conservation Actions In Place Classification Scheme v. 2.0, see <https://nc.iucnredlist.org/redlist/content/attachment_files/dec_2012_guidance_conservation_actions_in_place_classification_scheme.pdf> .

Current population trend: Current population trend refers to trends over a period of circa three years around the date of assessment; can be set by assessors as increasing, decreasing, stable, or unknown.

Data-sufficient species*:* Excludes species listed as Data Deficient on the IUCN Red List.

Harvest management plan: a plan exists for the species, or populations of the species, which sets out levels of (sustainable) harvest; assessors can select yes, no, or unknown. This information is not mandatory, only recommended.

International management or trade controls: Examples include CITES, Regional Fisheries Agreements, ICCAT, Marine Stewardship Council, Forestry Stewardship Council, Marine Aquarium Council, Phytosanitary Measures Agreement, and the US Endangered Species Act. (see <https://nc.iucnredlist.org/redlist/content/attachment_files/dec_2012_guidance_conservation_actions_in_place_classification_scheme.pdf>). Assessors can select yes, no, or unknown. This information is not mandatory, only recommended.

Recommended documentation: Recommended supporting information is not essential for a Red List assessment to be accepted for publication but is strongly encouraged for all assessments for taxa prioritized in the IUCN Red List Strategic Plan. See: <https://nc.iucnredlist.org/redlist/content/attachment_files/Required_and_Recommended_Supporting_Information_for_IUCN_Red_List_Assessments.pdf>.

Mandatory documentation: Supporting information that is mandatory for all Red List assessments before they can be accepted for publication. See: <https://nc.iucnredlist.org/redlist/content/attachment_files/Required_and_Recommended_Supporting_Information_for_IUCN_Red_List_Assessments.pdf>.

Threatened: Taxa listed as VU, EN, or CR on the IUCN Red List.

Threats classification scheme: Used by assessors to record past, ongoing and future threats to a taxon. Documenting major threats impacting a species is mandatory for species listed as EX, EW, threatened, and NT. For definitions, examples and guidance on the Threats Classification Scheme ver. 3.2, see: <https://nc.iucnredlist.org/redlist/content/attachment_files/dec_2012_guidance_threats_classification_scheme.pdf>

Threat impact score: A score calculated from a combination of threat scope, severity and timing. See Appendix S8.

Threat scope: The scope of the threat, affecting the whole (>90%); majority (50-90%); minority (<50%); or an unknown proportion of the population. This is only optional information to be provided in the Red List assessment.

Threat severity: The severity of the threat, given as: causing very rapid declines; rapid declines; slow, significant declines; causing/could cause fluctuations; negligible declines; no decline; or unknown. This is only optional information to be provided in the Red List assessment.

Threat timing: The timing of the threat given as: ongoing; past (unlikely to return); past (likely to return); future; or unknown. This is recommended information to be provided in the Red List assessment.

Use and trade classification scheme: Used by assessors to record how a taxon is utilized and what level of trade occurs for the taxon. This information is not mandatory, only recommended. For the General Use and Trade Classification Scheme (including the Non-Consumptive Use scheme) ver. 1.0, see <https://nc.iucnredlist.org/redlist/content/attachment_files/July_2020_Guidance_General_Use_and_Trade_Classification_Scheme.pdf>. See Appendix S3.

**Appendix S12.** Number and percentage of outdated (published prior to the year 2010) and up-to-date (published between 2010 and 2019) assessments for all extant, data-sufficient species and for the subset of species with adequate use and trade documentation.

| Taxonomic group^[[3]](#footnote-3)^ | < 2010^[[4]](#footnote-4)^ | | 2010 – 2019^[[5]](#footnote-5)^ | | Percentage outdated^[[6]](#footnote-6)^ | |
| --- | --- | --- | --- | --- | --- | --- |
|  | All | Used^[[7]](#footnote-7)^ | All | Used | All | Used |
| Aquatic | 1,320 | 348 | 5,283 | 2,326 | 20% | 13% |
| Selected bony fishes | 37 | 14 | 2,612 | 1,372 | 1% | 1% |
| Crustaceans | 624 | 35 | 1,125 | 228 | 36% | 13% |
| Cartilaginous Fishes | 239 | N/A | 447 | N/A | 35% | N/A |
| Corals | 420 | 299 | 223 | 182 | 65% | 62% |
| Cone Snails | 0 | 0 | 545 | 544 | 0 | 0 |
| Cephalopods | 0 | N/A | 331 | N/A | 0 | N/A |
| Terrestrial | 2,765 | 258 | 18,684 | 7,166 | 11% | 3% |
| Birds | 0 | 0 | 10,930 | 4,988 | 0 | 0 |
| Amphibians | 2,216 | 254 | 3,190 | 322 | 41% | 44% |
| Mammals | 539 | N/A | 4,358 | N/A | 11% | N/A |
| Selected dicots | 4 | 1 | 1,894 | 1,093 | 0 | 0 |
| Conifers | 0 | 0 | 602 | 458 | 0 | 0 |
| Cycads | 1 | 0 | 299 | 177 | 0 | 0 |
| Selected reptiles | 5 | 3 | 282 | 128 | 2% | 2% |
| Total | 4,085 | 606 | 26,838 | 9,492 | 13% | 13% |


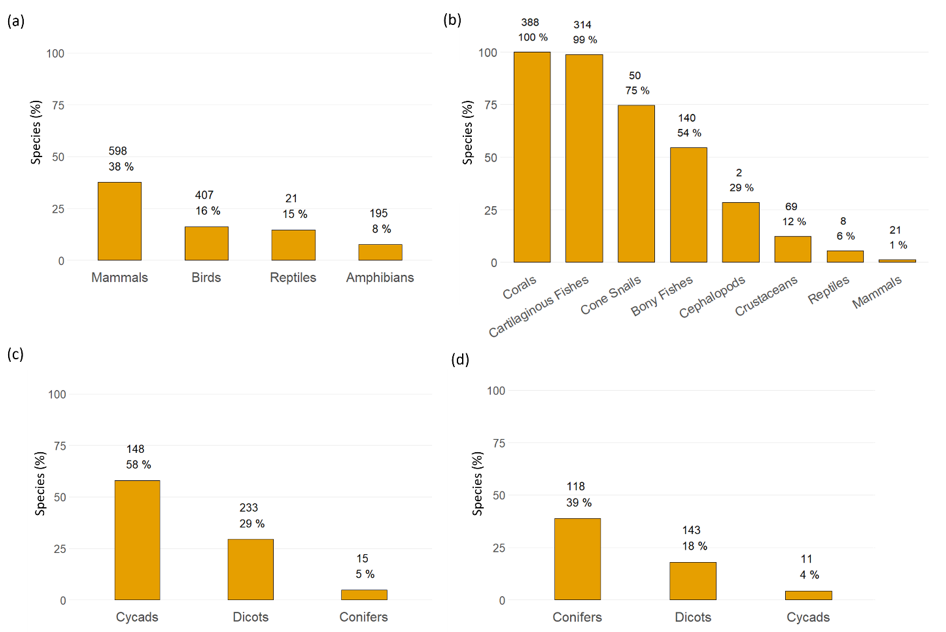


**Appendix S13.** Percentage of threatened and near threatened (NT) species with different forms of biological resource use documented as a threat on the International Union for Conservation of Nature Red List: (a) hunting and collection of terrestrial animals, (b) fishing and harvesting aquatic resources, (c) gathering terrestrial plants, and (d) logging and wood harvesting (numbers above bars, minimum number and percentage of species in each taxonomic group affected by the type of use). Some groups contain both terrestrial and aquatic species (e.g., 47 reptile species are affected by hunting and collection of terrestrial animals, and 10 by fishing and harvesting aquatic resources). We reassigned four species that were incorrectly coded on the Red List: *Glyptostrobus pensilis* (critically endangered, conifer), incorrectly coded as 5.4.1; *Euastacus brachythorax* (endangered, crayfish), incorrectly coded as 5.1.1 instead of 5.4.1; *Cambarus setosus* (NT, crayfish), incorrectly coded as 5.1.1 instead of 5.4.1; and *Conus boschorum* (NT, cone snail), incorrectly coded as 5.1.1 instead of 5.4.1. Bony fishes, dicotyledons (dicots) and reptiles include selected higher-level taxa (Appendix S1).


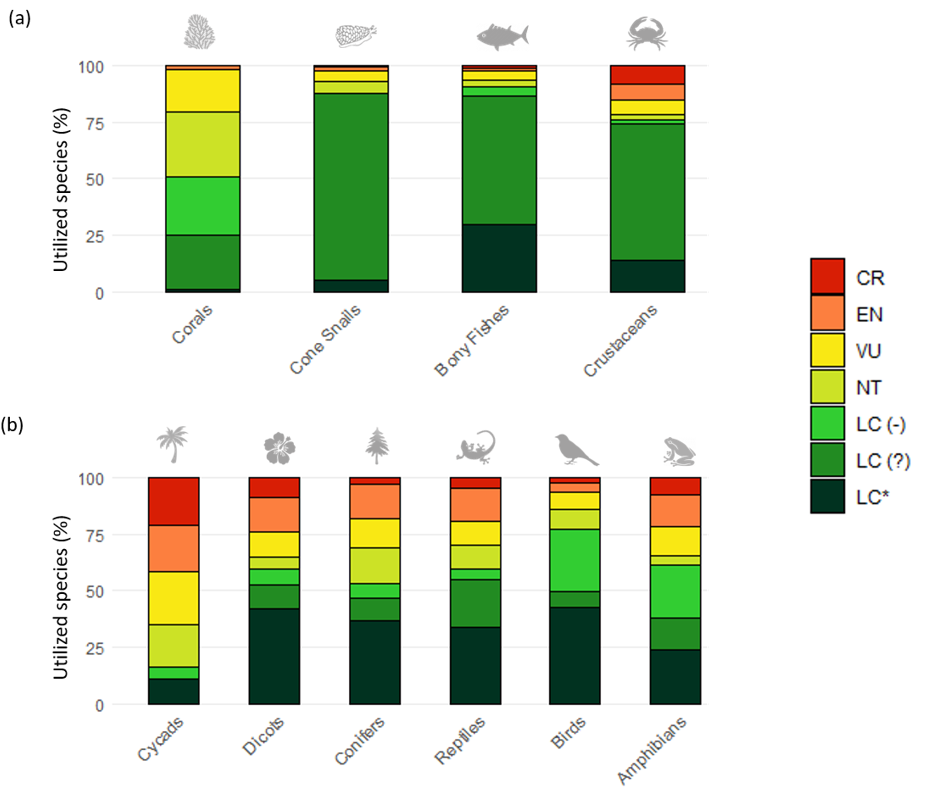


**Appendix S14.** Percentage of extant, data-sufficient species by International Union for Conservation of Nature Red List Category in (a) aquatic and (b) terrestrial groups subject to use and trade, excluding all species assessments published prior to 2010. (LC(-), least concern species with declining population trend; LC(?), least concern species with unknown population trend; LC(*), least concern species with stable or increasing population trend; NT, near threatened; VU, vulnerable; EN, endangered; CR, critically endangered). Being LC and having a declining population trend or being threatened and being subject to use and trade does not imply that use is a major threat. Bony fishes, dicotyledons (dicots), and reptiles include selected higher-level taxa (Appendix S1).


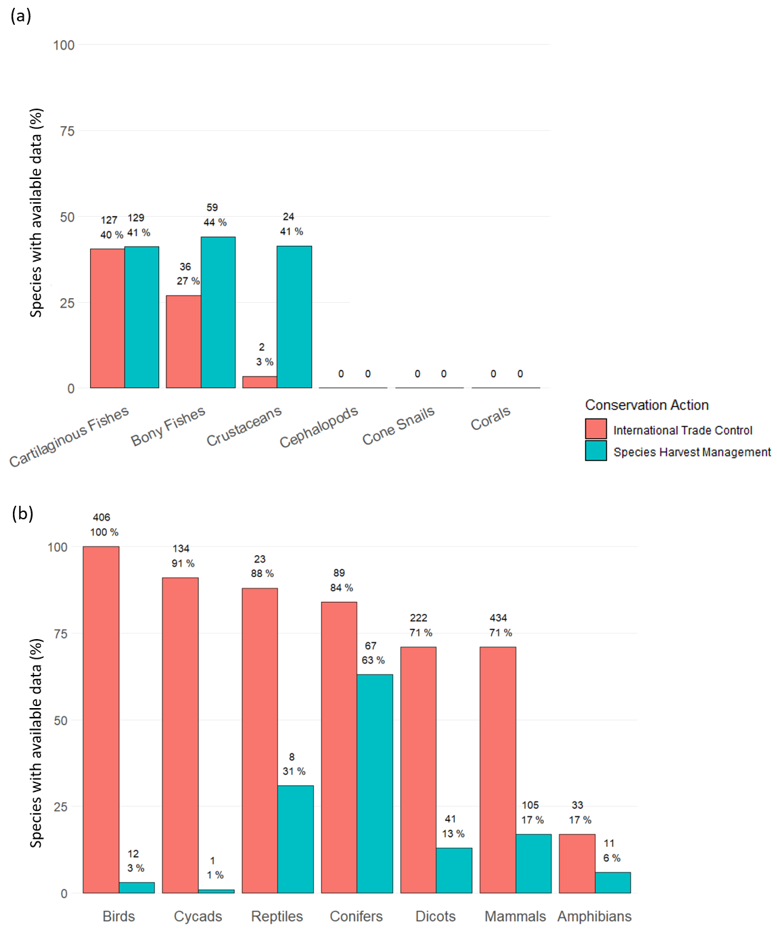


**Appendix S15.** Percentage of near threatened (NT) and threatened species in (a) aquatic and (b) terrestrial groups with biological resource use documented as a threat and with conservation actions data documented on the International Union for Conservation of Nature Red List (pink, number of species for which the international trade control field is coded as unknown, yes (meaning the species receive such management), or no (meaning the species does not receive such management), rather than left blank; blue, number of species for which the species management harvest plan field is coded as unknown, yes, or no; numbers above bars, species counts and percentages). Bony fishes, dicotyledons (dicots) and reptiles include selected higher-level taxa (Appendix S1).


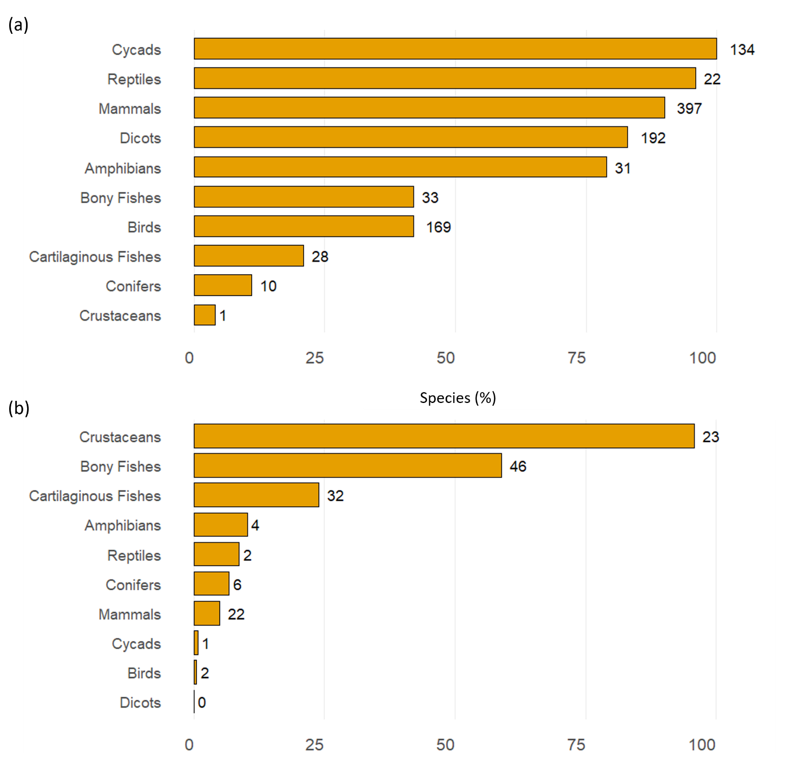
 **Appendix S16.** Percentage of near threatened (NT) and threatened species receiving (a) international trade controls and (b) targeted species harvest management with conservation actions data documented and biological resource use (minimum) documented as a threat on the International Union for Conservation of Nature Red List. No data are available for cephalopods, cone snails or corals (Appendix S15). Bony fishes, dicotyledons (dicots) and reptiles include selected higher-level taxa (Appendix S1).


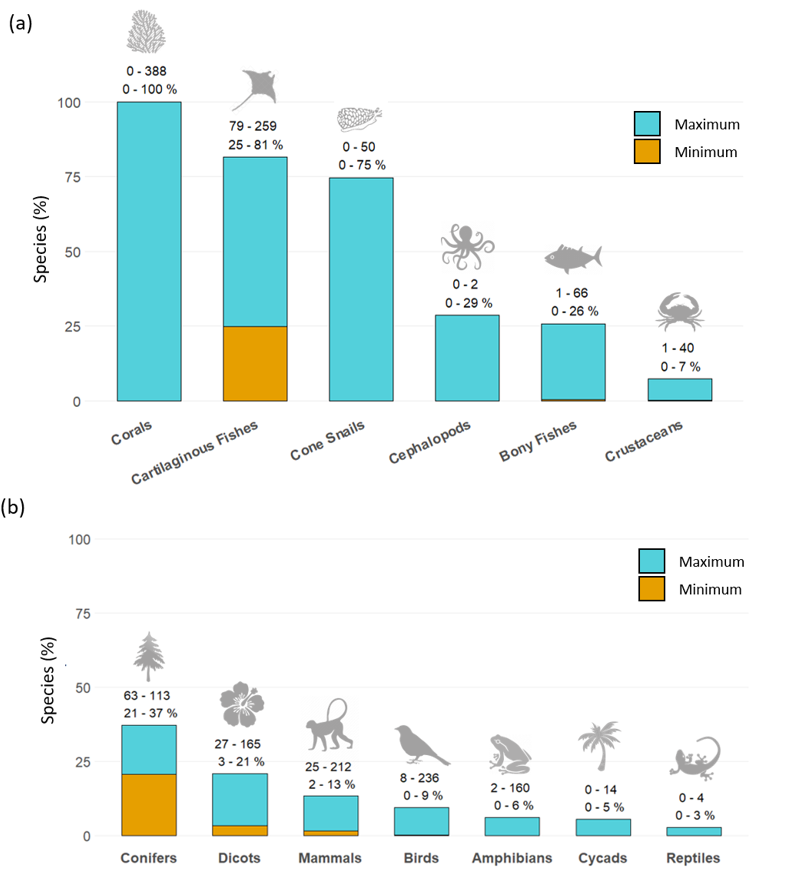


**Appendix S17.** Percentage of near threatened (NT) and threatened species in (a) aquatic and (b) terrestrial groups with biological resource use documented as a threat on the International Union for Conservation of Nature Red List and not receiving species conservation management interventions to directly address that use (orange, minimum number of species not receiving either international trade control or species harvest management actions, recorded as no; blue, maximum number of species for which management actions are either not documented for the species [field is left blank], or it is not known whether the species receives those actions [recorded as unknown]; numbers above bars, range of minimum to maximum species counts and percentages within each group). Bony fishes, dicotyledons (dicots) and reptiles include selected higher-level taxa (Appendix S1).

**Appendix S18.** Proposed amendments to the International Union for Conservation of Nature (IUCN)’s threats classification scheme class 5.3 and 5.4 following Salafsky et al. (2008 [Appendix S2]), to account for instances where scale of use is unknown but not motivation.

| Threats classification scheme 5. Biological resource use | | Suggested additions^[[8]](#footnote-8)^ |
| --- | --- | --- |
| 5.3 Logging & wood harvesting | |  |
|  | 5.3.1 Intentional use: subsistence and small scale (species being assessed is the target) |  |
|  | 5.3.2 Intentional use: large scale (species being assessed is the target) |  |
|  | 5.3.3 Intentional use: scale unknown (species being assessed is the target) [harvest] | new |
|  | 5.3.4 Unintentional effects: subsistence and small scale (species being assessed is not the target) |  |
|  | 5.3.5 Unintentional effects: large scale (species being assessed is not the target) |  |
|  | 5.3.6 Unintentional effects: scale unknown (species being assessed is not the target) [harvest] | new |
|  | 5.3.7 Motivation unknown or unrecorded |  |
| 5.4 Fishing & harvesting aquatic resources | |  |
|  | 5.4.1 Intentional use: subsistence and small scale (species being assessed is the target) |  |
|  | 5.4.2 Intentional use: large scale (species being assessed is the target) |  |
|  | 5.4.3 Intentional use: scale unknown (species being assessed is the target) [harvest] | new |
|  | 5.4.4 Unintentional effects: subsistence and small scale (species being assessed is not the target) |  |
|  | 5.4.5 Unintentional effects: large scale (species being assessed is not the target) |  |
|  | 5.4.6 Unintentional effects: scale unknown (species being assessed is not the target) [harvest] | new |
|  | 5.4.7 Persecution and control |  |
|  | 5.4.8 Motivation unknown or unrecorded |  |

**Appendix S19.** Supplementary discussion: recommendations for improving consistency and available information in use-related Red List data

In this paper, we propose a few recommendations (Table 2) that would help to reduce the proportion of used species – currently nearly half – for which we have no evidence as to whether intentional use is sustainable or not. As discussed in the main text, we do not make these recommendations lightly, given the delicate balance between expanding the taxonomic breadth of the Red List with the best possible supporting information and the need to undertake timely reassessments and the demand on the time and resources of individual assessors. We do however consider our recommendations achievable.

Recommendation one concerns coding of the threat category motivation unknown or unrecorded. In the current threats classification scheme, if the IUCN assessor does not know the scale of harvest (whether subsistence and small scale or large scale), their only option is to select the option for motivation unknown or unrecorded under the sections 5.3 (logging & wood harvesting) and 5.4. (fishing & harvesting aquatic resources), even though they are likely to know whether the use is intentional or unintentional. For instance, we found 1,519 amphibian species for which the threat of logging and wood harvesting was recorded, of which 1,187 were recorded under motivation unknown, but the motivation must have been unintentional as these species are impacted through the loss of forest, not through direct exploitation. Although our methodology excluded these cases from our analyses, we propose a modification to the IUCN’s threats classification scheme for assessors to indicate where the motivation is known, but the scale is not, to avoid these recording issues (Appendix S18).

Recommendation two is that data on timing, scope and severity of threats should be better documented as it would allow us to tease out more effectively where threat impacts are medium to high and bring greater precision to our results. For corals and cone snails, the threat of BRU is likely to be small in comparison with the impacts of bleaching and disease for corals (Carpenter et al. 2008), and urban pollution, tourism and coastal development for cone snails (Peters et al. 2013).

Further, it would be useful to better understand and quantify the degree to which species can be subject to some level of use without this resulting in them becoming threatened (i.e., impact is low, highly localized, negligible or no impact). This requires that the effects of BRU be more consistently recorded for LC species (recommendation three).

Recommendation four is for all assessments in the Red List Strategic Plan to comply with the recommended documentation requirements. One further advantage this would offer is that it would mean better availability of information on conservation actions in place and needed, respectively. Indeed, our results based on analysis of data in the IUCN’s conservation actions in place classification scheme are particularly constrained because data are available for a limited number of species. For example, only one taxonomic group (conifers) has more than half of its NT and threatened species with some documentation of whether a harvest management plan is in place (as opposed to being left blank). Whether international trade controls are in place is generally better documented than whether a harvest management plan is in place. This is likely because information on whether species are in a CITES Appendix or subject to some other policy controls is easier to obtain than on whether a harvest management plan is in place.

Finally, the addition of a check-box to indicate when the classification schemes for a given species assessment have been filled in at the recommended level, would be a powerful addition to the Red List documentation (recommendation five).

1. Taxonomic groups qualify for inclusion in this study according to criterion i if they have >40% of least concern (LC), threatened, and all extant, Data-sufficient species with at least one purpose of use recorded. Taxonomic groups meet criterion ii if the proportion of LC species with at least one purpose of use falls above or within the range of the proportion of species with use and trade documentation across the other IUCN Red List categories. [↑](#footnote-ref-1)
2. We excluded past threats which were deemed unlikely to return. Where severity or scope of threat is coded as unknown, we assign each a score of 2 (medium), meaning threats with unknown severity or scope were still analyzed if their timing was coded as future or likely to return. Consequently, for threats where both severity and scope were coded as unknown, our approach was precautionary in assuming the threat was at least medium impact; for threats where one of severity or scope are coded was unknown, and the other either slow or fluctuating, or negligible or low impact, our approach was evidentiary in assuming that the threat was not medium impact. [↑](#footnote-ref-2)
3. Taxonomic groups included in this study, classed as aquatic for primarily aquatic groups and terrestrial for primarily terrestrial groups. Bony fishes, dicotyledons (dicots), and reptiles include selected higher-level taxa, see Appendix S1 for detailed listing of taxonomic groups. Cephalopods, cartilaginous fishes, and mammals were excluded in analyses based on the use and trade classification scheme due to insufficient data (Appendix S5). [↑](#footnote-ref-3)
4. Number of Red List assessments of extant, data-sufficient species published before 2010. [↑](#footnote-ref-4)
5. Number of Red List assessments of extant, data-sufficient species published between 2010 and 2019. [↑](#footnote-ref-5)
6. Percentage of outdated assessments (published before 2010) formulated from the total numbers of assessments presented in Table 1 columns 1 (All) and 3 (Used). [↑](#footnote-ref-6)
7. Species with adequate use and trade documentation. [↑](#footnote-ref-7)
8. New denotes proposed amendments to the existing classification scheme. [↑](#footnote-ref-8)
