# Supplementary material for: Prevalence of sustainable and unsustainable use of wild species inferred from the IUCN Red List of Threatened Species
Source: Conserv Biol. 2021 Nov 17;36(2):e13844. doi: 10.1111/cobi.13844 (PMC9299080; doi:10.1111/cobi.13844)
Supplement: Supplementary file 2 — Supplementary material [file COBI-36-0-s002.pdf]

Supporting information

Appendix S1. Taxonomic description of species in comprehensively assessed groups of the International Union for Conservation of Nature (IUCN) Red List of Threatened Species.

Taxonomic group	Phylum	Class	Order	Family	Genus
Cephalopods	Mollusca	Cephalopoda*	All species		
Cone snails	Mollusca	Gastropoda	Neogastropoda	Conidae	Conus
Corals	Cnidaria	Hydrozoa	All species		
		Anthozoa	Helioporacea	All species	
			Scleractinia	Acroporidae	All species
				Agariciidae	All species
				Astrocoeniidae	All species
				Euphyllidae	All species
				Faviidae	All species
				Mussidae	All species
				Oculinidae	All species
				Pectiniidae	All species
				Pocilloporidae	All species
				Poritidae	All species
				Rhizangiidae	All species
				Siderastreidae	All species
				Trachyphylliidae	All species
				Turbinoliidae	All species
				Dendrophylliidae	Balanophyllia
					Duncanopsammia
					Heteropsammia
					Turbinaria
				Caryophylliidae	Heterocyathus
Cartilaginous fishes (sharks, rays, chimaeras)	Chordata	Chondrichthyes	All species		
Bony fishes (selected):					
Sturgeons	Chordata	Actinopterygii	Acipenseriformes	All species	
Tarpons & bonefishes			Albuliformes	All species	
			Elopiformes	All species	
Anchovies, sardines etc.			Clupeiformes	All species	

Groupers & wrasses			Perciformes	Epinephelidae	All species
				Labridae	All species
Tunas				Scombridae	All species
Billfishes				Istiophoridae	All species
				Xiphiidae	All species
Blennies				Blenniidae	All species
				Chaenopsidae	All species
				Clinidae	All species
				Dactyloscopidae	All species
				Labrisomidae	All species
				Tripterygiidae	All species
Seabreams				Sparidae	All species
				Centracanthidae	All species
Angelfishes				Pomacanthidae	All species
Butterflyfishes				Chaetodontidae	All species
Surgeonfishes				Acanthuridae	All species
Pufferfishes			Tetraodontiformes	Tetraodontidae	All species
Seahorses & pipefish			Syngnathiformes	Syngnathidae	All species
Trumpetfishes				Aulostomidae	All species
Shrimpfishes				Centriscidae	All species
Seamoths				Pegasidae	All species
Ghost pipefishes				Solenostomidae	All species
Cornetfishes			Gasterosteiformes	Fistulariidae	All species
Crustaceans (selected):					
Lobsters	Arthropoda	Malacostraca	Decapoda	Glypheidae	All species
				Polychelidae	All species
				Nephropidae	All species
				Enoplometopidae	All species
				Palinuridae	All species
				Scyllaridae	All species
Freshwater crayfishes				Astacidae	All species
				Cambaridae	All species
				Parastacidae	All species
Freshwater crabs				Trichodactylidae	All species
				Potamidae	All species
				Potamonautidae	All species
				Gecarcinucidae	All species
				Pseudothelphusidae	All species
Freshwater shrimps				Alpheidae	FW species only
				Atyidae	FW species only
				Desmocarididae	FW species only

				Euryrhynchidae	FW species only
				Palaemonidae	FW species only
				Typhlocarididae	FW species only
				Xiphocarididae	FW species only
Amphibians	Chordata	Amphibia	All species		
Birds	Chordata	Aves	All species		
Mammals	Chordata	Mammalia	All species		
Conifers	Tracheophyta	Pinopsida	All species		
Cycads		Cycadopsida	All species		
Dicotyledons (selected):					
Cacti	Tracheophyta	Magnoliopsida	Caryophyllales	Cactaceae	All species
Magnolias			Magnoliales	Magnoliaceae	All species
Birches			Fagales	Betulaceae	All species
Southern beeches				Nothofagaceae	All species
Teas			Ericales	Theaceae	All species
Reptiles (selected):					
Chameleons	Chordata	Reptilia	Squamata	Chamaeleonidae	All species
Sea snakes				Homalopsidae	All species
				Elapidae	Aipysurus
					Emydocephalus
					Ephalophis
					Hydrelaps
					Hydrophis
					Kerilia
					Kolpophis
					Laticauda
					Parahydrophis
					Thalassophis
				Acrochordidae	Acrochordus
				Natricidae	Anoplohydrus
Crocodiles & alligators			Crocodylia	All species	
Marine turtles			Testudines	Cheloniidae	All species
				Dermochelyidae	All species

*Nautiluses are not yet assessed

Appendix S2. The International Union for Conservation of Nature (IUCN)'s threats classification scheme class 5 following Salafsky et al. (2008)

Threats classification scheme 5. Biological resource use

5.1	Hunting & collecting terrestrial animals
	5.1.1 Intentional use (species being assessed is the target)
	5.1.2 Unintentional effects (species being assessed is not the target)
	5.1.3 Persecution and control
	5.1.4 Motivation unknown or unrecorded
5.2	Gathering terrestrial plants
	5.2.1 Intentional use (species being assessed is the target)
	5.2.2 Unintentional effects (species being assessed is not the target)
	5.2.3 Persecution and control
	5.2.4 Motivation unknown or unrecorded
5.3	Logging & wood harvesting
	5.3.1 Intentional use: subsistence and small scale (species being assessed is the target)
	5.3.2 Intentional use: large scale (species being assessed is the target)
	5.3.3 Unintentional effects: subsistence and small scale (species being assessed is not the target)
	5.3.4 Unintentional effects: large scale (species being assessed is not the target)
	5.3.5 Motivation unknown or unrecorded
5.4	Fishing & harvesting aquatic resources
	5.4.1 Intentional use: subsistence and small scale (species being assessed is the target)
	5.4.2 Intentional use: large scale (species being assessed is the target)
	5.4.3 Unintentional effects: subsistence and small scale (species being assessed is not the target)
	5.4.4 Unintentional effects: large scale (species being assessed is not the target)
	5.4.5 Persecution and control
	5.4.6 Motivation unknown or unrecorded

Appendix S3. The International Union for Conservation of Nature (IUCN)'s use and trade classification scheme. Analyses exclude use codes 16 through 18.

1. Food – human
2. Food – animal
3. Medicine – human & veterinary
4. Poisons
5. Manufacturing chemicals
6. Other chemicals
7. Fuels
8. Fibre
9. Construction or structural materials
10. Wearing apparel, accessories
11. Other household goods
12. Handicrafts, jewellery, etc.
13. Pets or display animals, horticulture
14. Research
15. Sport hunting and specimen collecting
16. Establishing ex situ production
17. Other (free text)
18. Unknown

Appendix S4. Supplementary methods: the main purposes of use of wild animal and plant species recorded in the International Union for Conservation of Nature (IUCN) Red List of Threatened Species

We investigated the prevalence of different purposes of use from the use and trade classification scheme recorded in the Red List of Threatened Species (Red List). Because completing the use and trade classification scheme is not mandatory for International Union for Conservation of Nature (IUCN) assessors, we investigated the prevalence of use and trade recording to decide which species groups to include in our analyses. We selected taxonomic groups for inclusion based on the following criteria: >40% of all extant, Data-sufficient species have at least one purpose of use coded (thus selecting taxonomic groups with high prevalence of use); or the proportion of least concern (LC) species with at least one purpose of use falls above or within the

range of the proportion of species with use and trade documentation across the other Red List categories (thus also selecting taxonomic groups where use and trade may be relatively low, but use and trade in LC species is coded to a similar level as that of species in other Red List categories). This limited our dataset to the following taxonomic groups which have adequate recording of use and trade: birds, amphibians, selected reptiles, cycads, conifers and selected dicots from the terrestrial group; and corals, selected bony fishes, crustaceans and cone snails from the aquatic species group (Appendix S5). We excluded mammals, cephalopods and cartilaginous fishes as they meet neither criterion, meaning within these groups there is either relatively little use, or Red List documentation of use is incomplete.

For each taxonomic group, we calculated the total number of species recorded as being used for at least one purpose in the use and trade classification scheme. However, we excluded use for the purpose of establishing ex-situ production (use code 16 [Appendix S3]), other (17), and unknown (18). Ex-situ production involves establishing captive populations for conservation breeding and translocation but also for ranching, farming or propagation purposes. It is not possible to distinguish these purposes without the IUCN assessors voluntarily providing information on harvest from captive and cultivated sources. Only eleven species (six cartilaginous fishes, four bony fishes, and one bird) in our analyses had an unknown use and trade category. We summarized the data as the percentage of species recorded for different types of use on the Red List.

Appendix S5. Proportion of extant, Data-sufficient species in each taxonomic group with at least one purpose of use documented on the International Union for Conservation of Nature (IUCN)'s use and trade classification scheme. *

Taxonomic group	Proportion LC	Proportion threatened	Proportion all species	LC in range	Criterion i	Criterion ii
Amphibians	0.12	0.09	0.11	Within	No	Yes
Birds	0.46	0.47	0.46	Within	Yes	Yes
Selected bony fishes	0.53	0.48	0.53	Within	Yes	Yes
Cartilaginous fishes	0.22	0.51	0.35	Below	No	No
Cephalopods	0.20	0.20	0.21	Below	No	No
Cone snails	1.00	0.98	1.00	Within	Yes	Yes
Conifers	0.82	0.69	0.76	Above	Yes	Yes
Corals	0.67	0.79	0.75	Within	Yes	Yes
Crustaceans	0.17	0.11	0.15	Above	No	Yes
Cycads	0.69	0.85	0.80	Below	Yes	No
Selected dicots	0.59	0.58	0.58	Within	Yes	Yes
Mammals	0.21	0.43	0.28	Below	No	No
Selected reptiles	0.53	0.41	0.46	Above	Yes	Yes

* Taxonomic groups qualify for inclusion in this study according to criterion i if they have >40% of least concern (LC), threatened, and all extant, Data-sufficient species with at least one purpose of use recorded. Taxonomic groups meet criterion ii if the proportion of LC species with at least one purpose of use falls above or within the range of the proportion of species with use and trade documentation across the other IUCN Red List categories.

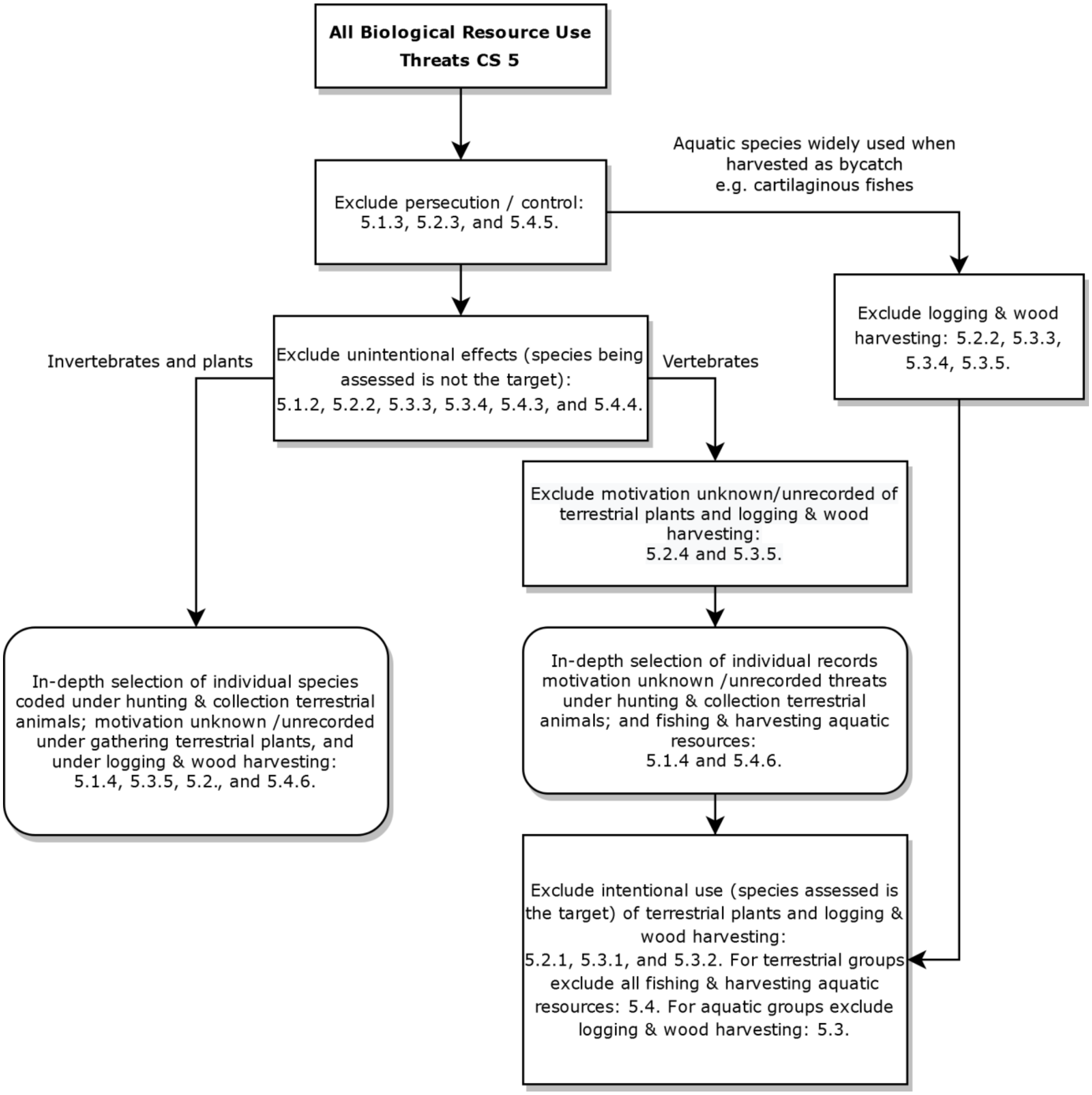

Appendix S6. Flow chart describing the process for including forms of biological resource use (based on the International Union for Conservation of Nature's threats classification scheme 5) that directly target the species in the taxonomic groups selected, focusing on intentional forms of use. In-depth selection entails checking a species' threat categories against detailed text information in the assessment records.

Appendix S7. Supplementary methods: wild species for which intentional use is having a negative impact on extinction risk

Because not all types of biological resource use (BRU) directly target species, we developed a decision-tree (Appendix S6) to remove threat types that are not relevant to analyses of sustainable and unsustainable direct and intentional use of species.

First, we discounted the threat of persecution or control, which does not represent intentional consumptive BRU. We excluded threats where the species was not the deliberate target of activity, here termed unintentional BRU, with the exception of aquatic species groups which are widely used even if unintentionally caught as bycatch (e.g., cartilaginous fishes; Dulvy et al. 2014). We also excluded records of vertebrates threatened by intentional gathering of plants, or by logging and wood harvesting, as these likely represent assessment errors and should have been coded as unintentional.

In addition to intentional and unintentional use, the IUCN’s threats classification scheme also allows for BRU to be recorded if the motivation behind use is unknown (i.e., the IUCN’s assessors do not know if use is intentionally targeting a species). While intentional gathering of terrestrial plants or logging and wood harvesting (threat codes 5.2.4 and 5.3.5) can threaten animals through habitat loss, they do not directly use animals and we excluded those records for animal groups. Where BRU was recorded with unknown motivation (codes 5.1.4 and 5.4.6 for animal species; codes 5.2.4 and 5.3.5 for plant species [Appendix S2]), we reviewed the detailed text of individual species’ assessments to determine whether these records should be excluded

from the analysis. For instance, Red List documentation records the Pygmy Slow Loris (*Nycticebus pygmaeus*) as threatened by intentional hunting and collecting of terrestrial animals (5.1.1), and by fishing and harvesting of aquatic resources for which the motivation is unknown or unrecorded (5.4.6). This terrestrial species is exploited for the pet trade and harvested for medicinal use, hunted as a food source, and experiences habitat loss due to agriculture. Its categorization under 5.4.6 likely represents a threat coding error and we excluded it, as no aquatic resource use is known to directly affect this terrestrial species. For some BRU threats with unknown motivation, we could not determine whether the threat directly targeted the species. We present this uncertainty in our results as a range where the minimum proportion includes all species with threats that could be conclusively determined as intentional (and hence more evidentiary), and the maximum proportion additionally includes species with use motivation unknown or unrecorded that may represent further cases of intentional BRU (and hence more precautionary). For groups that had no species with such threats, we present only the minimum.

We only included BRU if it had a major impact on species survival. The Red List uses a scoring system to derive threat impact, based on three elements: threat timing, categorized as past (unlikely to return), past (likely to return), ongoing, future, or unknown; threat scope, categorized as the percentage of the population affected by the threat, i.e., whole, >90%, majority, 50-90%, minority, <50%, or unknown; and threat severity, categorized as causing very rapid declines, rapid declines, slow and significant declines, causing or would cause fluctuations or negligible declines, or unknown. The IUCN uses this information to produce an overall threat impact score by summing the scores for timing, scope and severity for each threat impacting a species (IUCN

2020). While threat timing is recommended information to be provided in the Red List assessment (strongly encouraged but not essential to publication), severity and scope are optional. Because of this we utilized the impact score categorization with several amendments (Appendix S8). We treated threats classed as in the past but likely to return the same as future threats, awarding them a score of 1; we assigned a medium score of 2 to threats with either unknown or missing timing, severity and scope information. Subsequently, we excluded threats with an impact score signifying low, negligible or no impact, and retained medium to high impact threats (threat impact score >= 6). As an example, the amphibian *Herpele squalostoma* was threatened by intentional use (5.4.2, where the species is the target): this was an ongoing threat (timing) associated with a minority scope (<50% of population affected), causing negligible declines (severity). We calculated an impact score of 4 and excluded this threat from analysis. The Northern Fur Seal *Callorhinus ursinus* was threatened by large-scale intentional BRU (5.4.2) in the past (unlikely to return); a threat of majority scope, possibly causing fluctuations. This threat also yielded an impact score of 4 and was excluded. The main implication of our amended scoring is that it was precautionary in including threats where both severity and scope were marked as unknown (qualified as medium impact), but evidentiary in excluding threats if at least one of them was coded as negligible or minority.

Appendix S8. Scoring system for the impact of threats used in the current analysis, adapted from the International Union for Conservation of Nature (IUCN) Red List[*].

Timing:	Ongoing threat (+3)				Future or past, likely to return threat (long term) (+1)			
Severity: / Scope:	*Very rapid* (+3)	*Rapid or unknown* (+2)	*Slow or fluctuating* (+1)	*Negligible or no impact* (0)	*Very rapid* (+3)	*Rapid or Unknown* (+2)	*Slow or Fluctuating* (+1)	*Negligible or no impact* (0)
Whole (+3)	9	8	7	6	7	6	5	4
Majority or unknown (+2)	8	7	6	5	6	5	4	3
Minority (+1)	7	6	5	4	5	4	3	2

Additive impact scores:
8-9: High impact threat
6-7: Medium impact threat
3-5: Low impact threat
0-2: Negligible or no impact threat

[*] We excluded past threats which were deemed unlikely to return. Where severity or scope of threat is coded as unknown, we assign each a score of 2 (medium), meaning threats with unknown severity or scope were still analyzed if their timing was coded as future or likely to return. Consequently, for threats where both severity and scope were coded as unknown, our approach was precautionary in assuming the threat was at least medium impact; for threats where one of severity or scope are coded was unknown, and the other either slow or fluctuating, or negligible or low impact, our approach was evidentiary in assuming that the threat was not medium impact.

Appendix S9. Threatened or near threatened species with at least one purpose of use or trade recorded that are not impacted by major, intentional biological resource use, and have stable or increasing population trends at the time of assessment.

**Taxonomic group**	**Scientific name**	**Red List Category**	**Population trend**
Amphibians	*Leptobrachium ailaonicum*	Near Threatened	Stable
Amphibians	*Atelopus flavescens*	Vulnerable	Stable
Amphibians	*Eurycea rathbuni*	Vulnerable	Stable
Amphibians	*Lyciasalamandra helverseni*	Vulnerable	Stable
Amphibians	*Lyciasalamandra luschani*	Vulnerable	Stable
Amphibians	*Lyciasalamandra atifi*	Endangered	Stable
Birds	*Accipiter collaris*	Near Threatened	Stable
Birds	*Accipiter poliogaster*	Near Threatened	Increasing
Birds	*Acrocephalus rodericanus*	Near Threatened	Increasing
Birds	*Actinodura sodangorum*	Near Threatened	Stable
Birds	*Anas chlorotis*	Near Threatened	Increasing
Birds	*Buteo solitarius*	Near Threatened	Stable
Birds	*Callaeas wilsoni*	Near Threatened	Increasing
Birds	*Caprimulgus phalaena*	Near Threatened	Stable
Birds	*Charadrius aquilonius*	Near Threatened	Increasing
Birds	*Charadrius melodus*	Near Threatened	Increasing
Birds	*Charadrius pallidus*	Near Threatened	Stable
Birds	*Chloebia gouldiae*	Near Threatened	Stable
Birds	*Cynanthus lawrencei*	Near Threatened	Stable
Birds	*Dendrocopos owstoni*	Near Threatened	Stable
Birds	*Dendropicos stierlingi*	Near Threatened	Stable
Birds	*Ducula whartoni*	Near Threatened	Stable
Birds	*Egretta rufescens*	Near Threatened	Increasing
Birds	*Elanus scriptus*	Near Threatened	Stable
Birds	*Erythrura coloria*	Near Threatened	Stable
Birds	*Estrilda poliopareia*	Near Threatened	Stable
Birds	*Eudyptes schlegeli*	Near Threatened	Stable
Birds	*Euplectes jacksoni*	Near Threatened	Stable
Birds	*Foudia flavicans*	Near Threatened	Increasing
Birds	*Fringilla teydea*	Near Threatened	Increasing
Birds	*Garrulax nuchalis*	Near Threatened	Stable
Birds	*Gyps himalayensis*	Near Threatened	Stable
Birds	*Hemiphaga novaeseelandiae*	Near Threatened	Increasing
Birds	*Hypsipetes borbonicus*	Near Threatened	Increasing
Birds	*Larus atlanticus*	Near Threatened	Stable

Birds	*Larvivora komadori*	Near Threatened	Stable
Birds	*Megascops marshalli*	Near Threatened	Stable
Birds	*Microcarbo coronatus*	Near Threatened	Stable
Birds	*Oxyura australis*	Near Threatened	Stable
Birds	*Parotia wahnesi*	Near Threatened	Stable
Birds	*Phalcoboenus australis*	Near Threatened	Stable
Birds	*Philesturnus carunculatus*	Near Threatened	Increasing
Birds	*Philesturnus rufusater*	Near Threatened	Increasing
Birds	*Phoebastria immutabilis*	Near Threatened	Stable
Birds	*Phoebastria nigripes*	Near Threatened	Increasing
Birds	*Polytelis alexandrae*	Near Threatened	Stable
Birds	*Psittinus abbotti*	Near Threatened	Stable
Birds	*Pyrilia caica*	Near Threatened	Stable
Birds	*Pyrrhura devillei*	Near Threatened	Stable
Birds	*Speculanas specularis*	Near Threatened	Stable
Birds	*Streptopelia reichenowi*	Near Threatened	Stable
Birds	*Thalasseus elegans*	Near Threatened	Stable
Birds	*Todiramphus pelewensis*	Near Threatened	Stable
Birds	*Amazona arausiaca*	Vulnerable	Increasing
Birds	*Amazona guildingii*	Vulnerable	Increasing
Birds	*Anas albogularis*	Vulnerable	Stable
Birds	*Anas aucklandica*	Vulnerable	Stable
Birds	*Anthropoides paradiseus*	Vulnerable	Stable
Birds	*Ardenna bulleri*	Vulnerable	Stable
Birds	*Branta sandvicensis*	Vulnerable	Increasing
Birds	*Buteo galapagoensis*	Vulnerable	Stable
Birds	*Capito wallacei*	Vulnerable	Stable
Birds	*Charadrius sanctaehelenae*	Vulnerable	Increasing
Birds	*Coracopsis barklyi*	Vulnerable	Stable
Birds	*Cyanoramphus unicolor*	Vulnerable	Stable
Birds	*Diomedea epomophora*	Vulnerable	Stable
Birds	*Eunymphicus cornutus*	Vulnerable	Increasing
Birds	*Eunymphicus uvaeensis*	Vulnerable	Increasing
Birds	*Falco araeus*	Vulnerable	Stable
Birds	*Falco hypoleucos*	Vulnerable	Stable
Birds	*Fregata aquila*	Vulnerable	Stable
Birds	*Fulica alai*	Vulnerable	Stable
Birds	*Grus monacha*	Vulnerable	Increasing
Birds	*Hemiphaga chathamensis*	Vulnerable	Increasing
Birds	*Icterus oberi*	Vulnerable	Stable
Birds	*Leucocarbo campbelli*	Vulnerable	Stable
Birds	*Leucocarbo colensoi*	Vulnerable	Stable
Birds	*Leucocarbo ranfurlyi*	Vulnerable	Stable

Birds	*Lonchura vana*	Vulnerable	Stable
Birds	*Megapodius pritchardii*	Vulnerable	Increasing
Birds	*Nannopterum harrisi*	Vulnerable	Stable
Birds	*Nesoenas mayeri*	Vulnerable	Stable
Birds	*Ninox natalis*	Vulnerable	Stable
Birds	*Odontophorus dialeucos*	Vulnerable	Stable
Birds	*Phoebastria albatrus*	Vulnerable	Increasing
Birds	*Phoenicoparrus andinus*	Vulnerable	Stable
Birds	*Pica nutalli*	Vulnerable	Stable
Birds	*Poicephalus robustus*	Vulnerable	Stable
Birds	*Procellaria parkinsoni*	Vulnerable	Stable
Birds	*Psittacula eques*	Vulnerable	Increasing
Birds	*Pterodroma axillaris*	Vulnerable	Increasing
Birds	*Pterodroma deserta*	Vulnerable	Stable
Birds	*Pterodroma solandri*	Vulnerable	Increasing
Birds	*Pyrrhula murina*	Vulnerable	Stable
Birds	*Pyrrhura perlata*	Vulnerable	Stable
Birds	*Scolopax mira*	Vulnerable	Stable
Birds	*Thalassarche eremita*	Vulnerable	Stable
Birds	*Touit huetii*	Vulnerable	Stable
Birds	*Alopecoenas rubescens*	Endangered	Stable
Birds	*Copsychus sechellarum*	Endangered	Increasing
Birds	*Cyclopsitta coxeni*	Endangered	Stable
Birds	*Foudia aldabrana*	Endangered	Increasing
Birds	*Fringilla polatzeki*	Endangered	Increasing
Birds	*Grus americana*	Endangered	Increasing
Birds	*Hypotaenidia sylvestris*	Endangered	Stable
Birds	*Junco insularis*	Endangered	Increasing
Birds	*Nipponia nippon*	Endangered	Increasing
Birds	*Ognorhynchus icterotis*	Endangered	Increasing
Birds	*Papasula abbotti*	Endangered	Stable
Birds	*Platalea minor*	Endangered	Increasing
Birds	*Porphyrio hochstetteri*	Endangered	Stable
Birds	*Pterodroma cahow*	Endangered	Increasing
Birds	*Pterodroma madeira*	Endangered	Stable
Birds	*Rhyticeros narcondami*	Endangered	Stable
Birds	*Xenoperdix udzungwensis*	Endangered	Stable
Birds	*Amazilia alfaroana*	Critically Endangered	Stable
Birds	*Aythya innotata*	Critically Endangered	Stable
Birds	*Cyanoramphus malherbi*	Critically Endangered	Stable
Birds	*Gymnogyps californianus*	Critically Endangered	Increasing
Birds	*Hypotaenidia owstoni*	Critically Endangered	Increasing
Birds	*Pterodroma magentae*	Critically Endangered	Increasing

Birds	*Strigops habroptila*	Critically Endangered	Increasing
Birds	*Vini ultramarina*	Critically Endangered	Stable
Bony Fishes	*Centropyge nahackyi*	Near Threatened	Stable
Bony Fishes	*Holacanthus limbaughi*	Near Threatened	Stable
Bony Fishes	*Holacanthus clarionensis*	Vulnerable	Stable
Cone Snails	*Conus kersteni*	Near Threatened	Stable
Cone Snails	*Conus fontonae*	Vulnerable	Stable
Cone Snails	*Conus teodorae*	Vulnerable	Stable
Cone Snails	*Conus xicoi*	Vulnerable	Stable
Conifers	*Abies bracteata*	Near Threatened	Stable
Conifers	*Abies kawakamii*	Near Threatened	Stable
Conifers	*Agathis atropurpurea*	Near Threatened	Stable
Conifers	*Agathis microstachya*	Near Threatened	Stable
Conifers	*Austrocedrus chilensis*	Near Threatened	Increasing
Conifers	*Chamaecyparis lawsoniana*	Near Threatened	Increasing
Conifers	*Cryptomeria japonica*	Near Threatened	Stable
Conifers	*Halocarpus kirkii*	Near Threatened	Stable
Conifers	*Libocedrus bidwillii*	Near Threatened	Stable
Conifers	*Libocedrus plumosa*	Near Threatened	Increasing
Conifers	*Pherosphaera hookeriana*	Near Threatened	Stable
Conifers	*Pinus balfouriana*	Near Threatened	Stable
Conifers	*Pinus jaliscana*	Near Threatened	Stable
Conifers	*Amentotaxus formosana*	Vulnerable	Stable
Conifers	*Callitris oblonga*	Vulnerable	Stable
Conifers	*Cupressus macrocarpa*	Vulnerable	Stable
Conifers	*Prumnopitys ladei*	Vulnerable	Stable
Conifers	*Thuja sutchuenensis*	Endangered	Increasing
Conifers	*Juniperus bermudiana*	Critically Endangered	Increasing
Cycads	*Encephalartos septentrionalis*	Near Threatened	Stable
Cycads	*Macrozamia longispina*	Near Threatened	Stable
Cycads	*Zamia pseudomonticola*	Near Threatened	Stable
Cycads	*Cycas semota*	Vulnerable	Stable
Dicots	*Cleistocactus acanthurus*	Near Threatened	Stable
Dicots	*Echinocactus parryi*	Near Threatened	Stable
Dicots	*Echinocereus websterianus*	Near Threatened	Stable
Dicots	*Mammillaria boolii*	Near Threatened	Stable
Dicots	*Pachycereus lepidanthus*	Near Threatened	Stable
Dicots	*Parodia columnaris*	Near Threatened	Stable
Dicots	*Rebutia arenacea*	Near Threatened	Stable
Dicots	*Rhipsalis olivifera*	Near Threatened	Stable
Dicots	*Cephalocereus nizandensis*	Vulnerable	Stable
Dicots	*Discocactus horstii*	Vulnerable	Stable
Dicots	*Gymnocalycium marianae*	Vulnerable	Stable

Dicots	*Mammillaria multidigitata*	Vulnerable	Stable
Dicots	*Mammillaria tayloriorum*	Vulnerable	Stable
Dicots	*Neobuxbaumia polylopha*	Vulnerable	Stable
Dicots	*Schlumbergera microsphaerica*	Vulnerable	Stable
Dicots	*Cleistocactus sulcifer*	Endangered	Stable
Reptiles	*Bradypodion dracomontanum*	Near Threatened	Stable
Reptiles	*Furcifer timoni*	Near Threatened	Stable
Reptiles	*Kinyongia oxyrhina*	Near Threatened	Stable

Appendix S10. Summary of analyses undertaken

We provide a short-hand summary of the main analyses undertaken in this paper, which correspond to the numbers at the bottom of Table 1.

1. The main purposes of use of wild animal and plant species on the Red List

Estimated as: the total number of species recorded as being used for at least one purpose of use as the percentage of extant, data-sufficient species recorded for different types of use (Fig. 1)

2. Wild species for which intentional use is having a negative impact on extinction risk (i.e., for which BRU is documented as a major threat), estimated from among:

a. all extant, data-sufficient species with at least one purpose of use coded (sensu analysis 1);
b. all NT and threatened species (from among all 13 taxonomic groups comprehensively assessed; Fig. 2, Appendix S13)

3. Wild species for which intentional BRU is not having a negative impact on extinction risk. Estimated from the number of extant, Data-sufficient species recorded as being subject to some form of use or trade that:

a. are currently LC;
b. currently LC and not declining (i.e., have either stable or increasing current population trends at the time of assessment; Fig. 3);
c. are threatened or NT and are not documented as having intentional BRU as a major threat and have stable or increasing population trends at the time of assessment.

4. Conservation actions in place or lacking for utilized wild species. Estimated from the number of NT and threatened species that are adversely affected by biological resource use and:

a. are coded as receiving either one or both of international trade controls and / or harvest management actions (Fig. 4);
b. are not receiving either of these conservation management actions.

Appendix S11. Glossary of Red List-related terms used in the manuscript

Biological resource use: threats from consumptive use of “wild” biological resources including deliberate and unintentional harvesting effects; also, persecution or control of specific species (Salafsky et al. 2008). Part of the IUCN-Conservation Measures Partnership (CMP) classification of direct threats to biodiversity and the IUCN Red List Threat Classification Scheme (Class 5). See Appendix S2.

Comprehensively assessed / comprehensive assessment: taxonomic groups that include at least 150 species, of which >80% have been assessed (IUCN 2020).

Conservation Actions In Place Classification Scheme*:* Used by assessors to record what conservation actions are already in place for a taxon. This information is not mandatory, only recommended. For definitions, examples and guidance on the Conservation Actions In Place Classification Scheme v. 2.0, see https://nc.iucnredlist.org/redlist/content/attachment_files/dec_2012_guidance_conservation_actions_in_place_classification_scheme.pdf .

Current population trend: Current population trend refers to trends over a period of circa three years around the date of assessment; can be set by assessors as increasing, decreasing, stable, or unknown.

Data-sufficient species*:* Excludes species listed as Data Deficient on the IUCN Red List.

Harvest management plan: a plan exists for the species, or populations of the species, which sets out levels of (sustainable) harvest; assessors can select yes, no, or unknown. This information is not mandatory, only recommended.

International management or trade controls: Examples include CITES, Regional Fisheries Agreements, ICCAT, Marine Stewardship Council, Forestry Stewardship Council, Marine Aquarium Council, Phytosanitary Measures Agreement, and the US Endangered Species Act. (see https://nc.iucnredlist.org/redlist/content/attachment_files/dec_2012_guidance_conservation_actions_in_place_classification_scheme.pdf). Assessors can select yes, no, or unknown. This information is not mandatory, only recommended.

Recommended documentation: Recommended supporting information is not essential for a Red List assessment to be accepted for publication but is strongly encouraged for all assessments for taxa prioritized in the IUCN Red List Strategic Plan. See: https://nc.iucnredlist.org/redlist/content/attachment_files/Required_and_Recommended_Supporting_Information_for_IUCN_Red_List_Assessments.pdf.

Mandatory documentation: Supporting information that is mandatory for all Red List assessments before they can be accepted for publication. See: https://nc.iucnredlist.org/redlist/content/attachment_files/Required_and_Recommended_Supporting_Information_for_IUCN_Red_List_Assessments.pdf.

Threatened: Taxa listed as VU, EN, or CR on the IUCN Red List.

Threats classification scheme: Used by assessors to record past, ongoing and future threats to a taxon. Documenting major threats impacting a species is mandatory for species listed as EX, EW, threatened, and NT. For definitions, examples and guidance on the Threats Classification Scheme ver. 3.2, see: https://nc.iucnredlist.org/redlist/content/attachment_files/dec_2012_guidance_threats_classification_scheme.pdf

Threat impact score: A score calculated from a combination of threat scope, severity and timing. See Appendix S8.

Threat scope: The scope of the threat, affecting the whole (>90%); majority (50-90%); minority (<50%); or an unknown proportion of the population. This is only optional information to be provided in the Red List assessment.

Threat severity: The severity of the threat, given as: causing very rapid declines; rapid declines; slow, significant declines; causing/could cause fluctuations; negligible declines; no decline; or unknown. This is only optional information to be provided in the Red List assessment.

Threat timing: The timing of the threat given as: ongoing; past (unlikely to return); past (likely to return); future; or unknown. This is recommended information to be provided in the Red List assessment.

Use and trade classification scheme: Used by assessors to record how a taxon is utilized and what level of trade occurs for the taxon. This information is not mandatory, only recommended. For the General Use and Trade Classification Scheme (including the Non-Consumptive Use scheme) ver. 1.0, see https://nc.iucnredlist.org/redlist/content/attachment_files/July_2020_Guidance_General_Use_and_Trade_Classification_Scheme.pdf. See Appendix S3.

Appendix S12. Number and percentage of outdated (published prior to the year 2010) and up-to-date (published between 2010 and 2019) assessments for all extant, data-sufficient species and for the subset of species with adequate use and trade documentation.

	< 2010[†]		2010 – 2019[‡]		Percentage outdated[§]	
Taxonomic group[*]	All	Used[**]	All	Used	All	Used
Aquatic	1,320	348	5,283	2,326	20%	13%
Selected bony fishes	37	14	2,612	1,372	1%	1%
Crustaceans	624	35	1,125	228	36%	13%
Cartilaginous Fishes	239	N/A	447	N/A	35%	N/A
Corals	420	299	223	182	65%	62%
Cone Snails	0	0	545	544	0	0
Cephalopods	0	N/A	331	N/A	0	N/A
Terrestrial	2,765	258	18,684	7,166	11%	3%
Birds	0	0	10,930	4,988	0	0
Amphibians	2,216	254	3,190	322	41%	44%
Mammals	539	N/A	4,358	N/A	11%	N/A
Selected dicots	4	1	1,894	1,093	0	0
Conifers	0	0	602	458	0	0
Cycads	1	0	299	177	0	0
Selected reptiles	5	3	282	128	2%	2%
Total	4,085	606	26,838	9,492	13%	13%

[*] Taxonomic groups included in this study, classed as aquatic for primarily aquatic groups and terrestrial for primarily terrestrial groups. Bony fishes, dicotyledons (dicots), and reptiles include selected higher-level taxa, see Appendix S1 for detailed listing of taxonomic groups. Cephalopods, cartilaginous fishes, and mammals were excluded in analyses based on the use and trade classification scheme due to insufficient data (Appendix S5).

[†] Number of Red List assessments of extant, data-sufficient species published before 2010.

[‡] Number of Red List assessments of extant, data-sufficient species published between 2010 and 2019.

[§] Percentage of outdated assessments (published before 2010) formulated from the total numbers of assessments presented in Table 1 columns 1 (All) and 3 (Used).

[**] Species with adequate use and trade documentation.

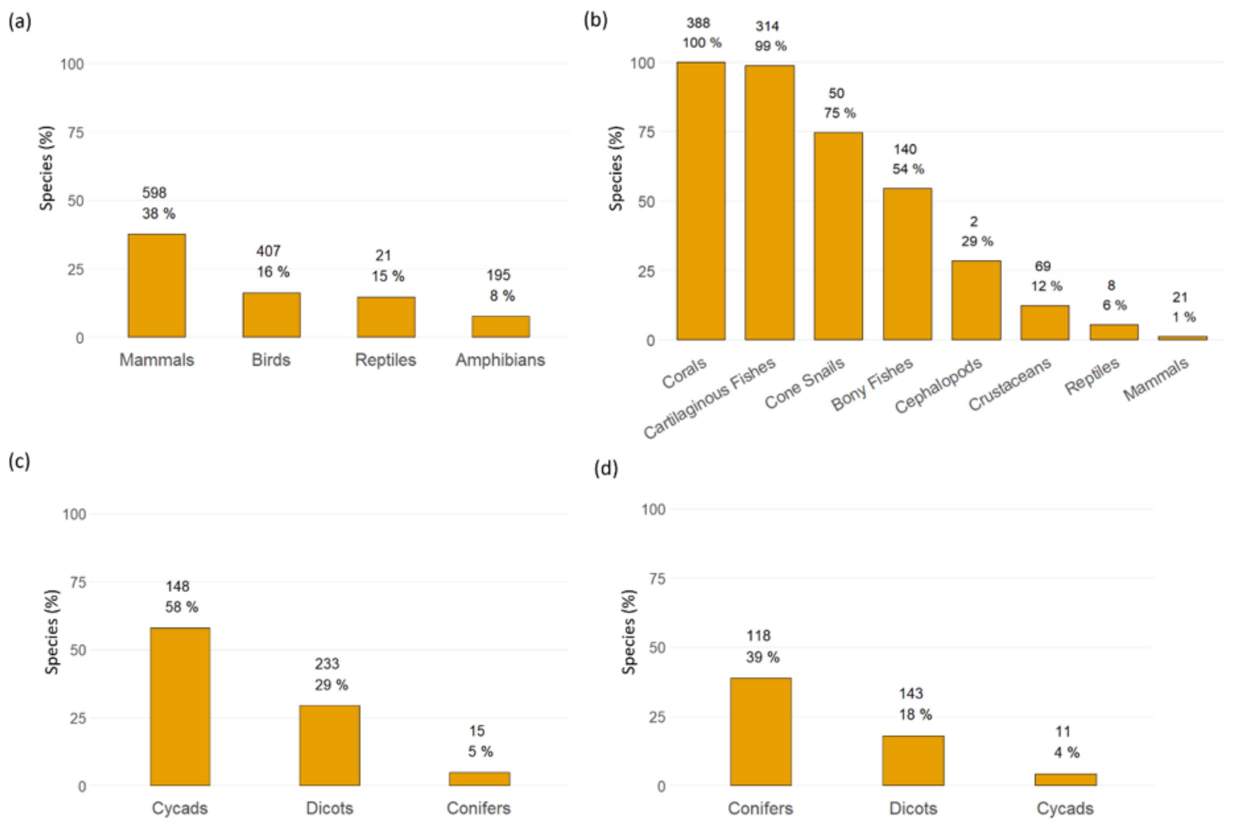

Appendix S13. Percentage of threatened and near threatened (NT) species with different forms of biological resource use documented as a threat on the International Union for Conservation of Nature Red List: (a) hunting and collection of terrestrial animals, (b) fishing and harvesting aquatic resources, (c) gathering terrestrial plants, and (d) logging and wood harvesting (numbers above bars, minimum number and percentage of species in each taxonomic group affected by the type of use). Some groups contain both terrestrial and aquatic species (e.g., 47 reptile species are affected by hunting and collection of terrestrial animals, and 10 by fishing and harvesting aquatic resources). We reassigned four species that were incorrectly coded on the Red List: *Glyptostrobus pensilis* (critically endangered, conifer), incorrectly coded as 5.4.1; *Euastacus brachythorax* (endangered, crayfish), incorrectly coded as 5.1.1 instead of 5.4.1; *Cambarus setosus* (NT, crayfish), incorrectly coded as 5.1.1 instead of 5.4.1; and *Conus boschorum* (NT,

cone snail), incorrectly coded as 5.1.1 instead of 5.4.1. Bony fishes, dicotyledons (dicots) and reptiles include selected higher-level taxa (Appendix S1).

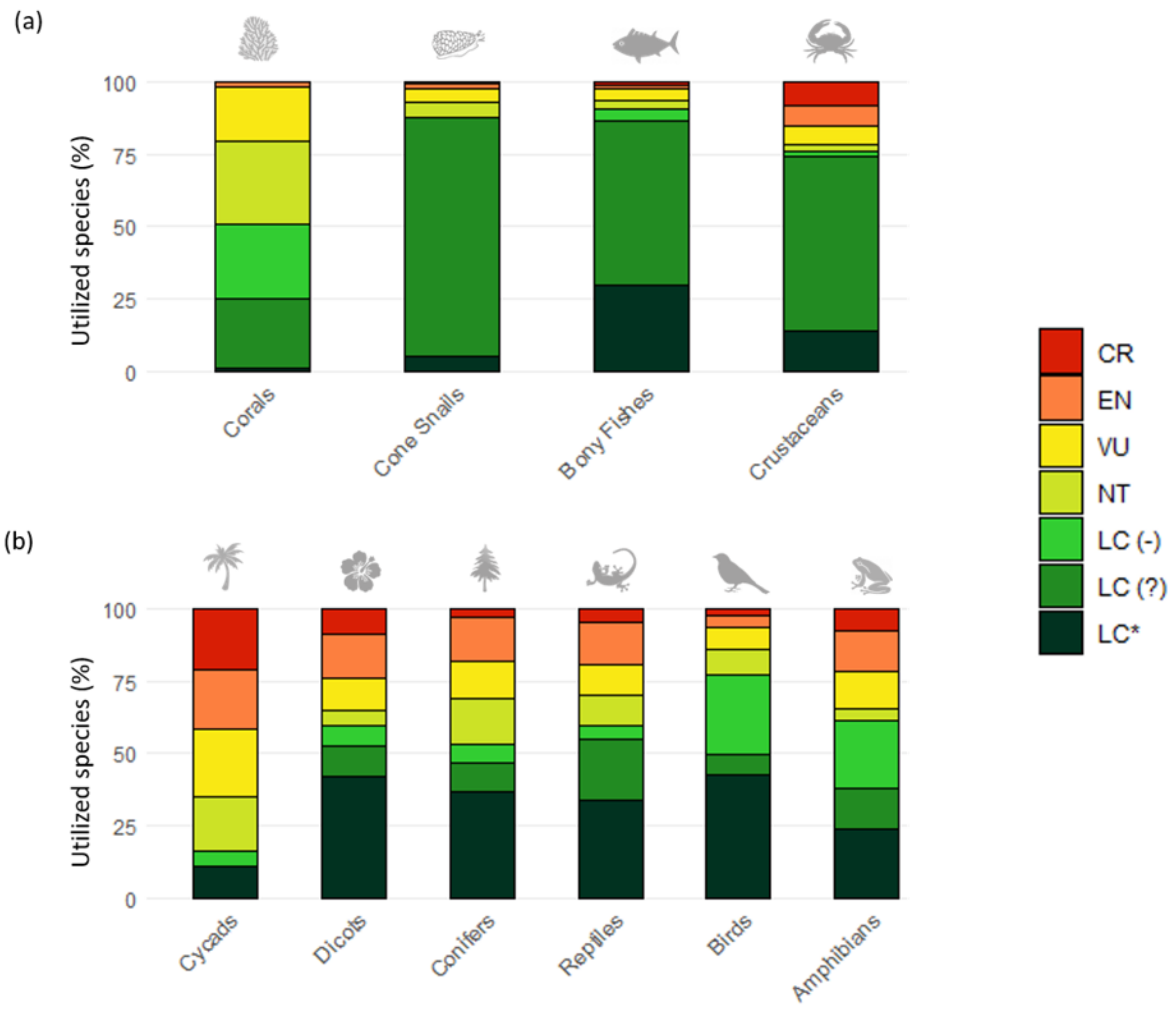

Appendix S14. Percentage of extant, data-sufficient species by International Union for Conservation of Nature Red List Category in (a) aquatic and (b) terrestrial groups subject to use and trade, excluding all species assessments published prior to 2010. (LC(-), least concern species with declining population trend; LC(?), least concern species with unknown population

trend; LC(*), least concern species with stable or increasing population trend; NT, near threatened; VU, vulnerable; EN, endangered; CR, critically endangered). Being LC and having a declining population trend or being threatened and being subject to use and trade does not imply that use is a major threat. Bony fishes, dicotyledons (dicots), and reptiles include selected higher-level taxa (Appendix S1).

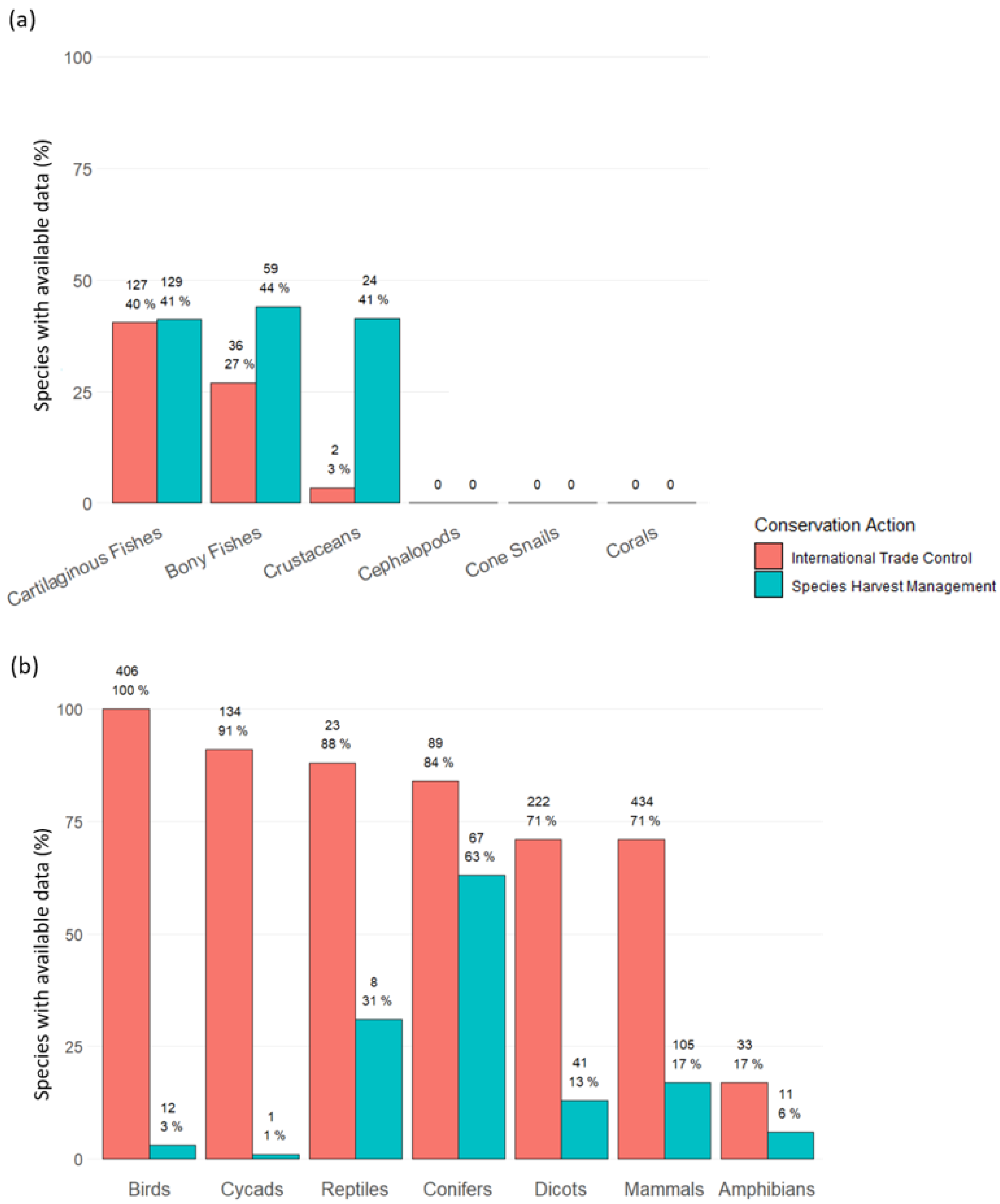

Appendix S15. Percentage of near threatened (NT) and threatened species in (a) aquatic and (b) terrestrial groups with biological resource use documented as a threat and with conservation

actions data documented on the International Union for Conservation of Nature Red List (pink, number of species for which the international trade control field is coded as unknown, yes (meaning the species receive such management), or no (meaning the species does not receive such management), rather than left blank; blue, number of species for which the species management harvest plan field is coded as unknown, yes, or no; numbers above bars, species counts and percentages). Bony fishes, dicotyledons (dicots) and reptiles include selected higher-level taxa (Appendix S1).

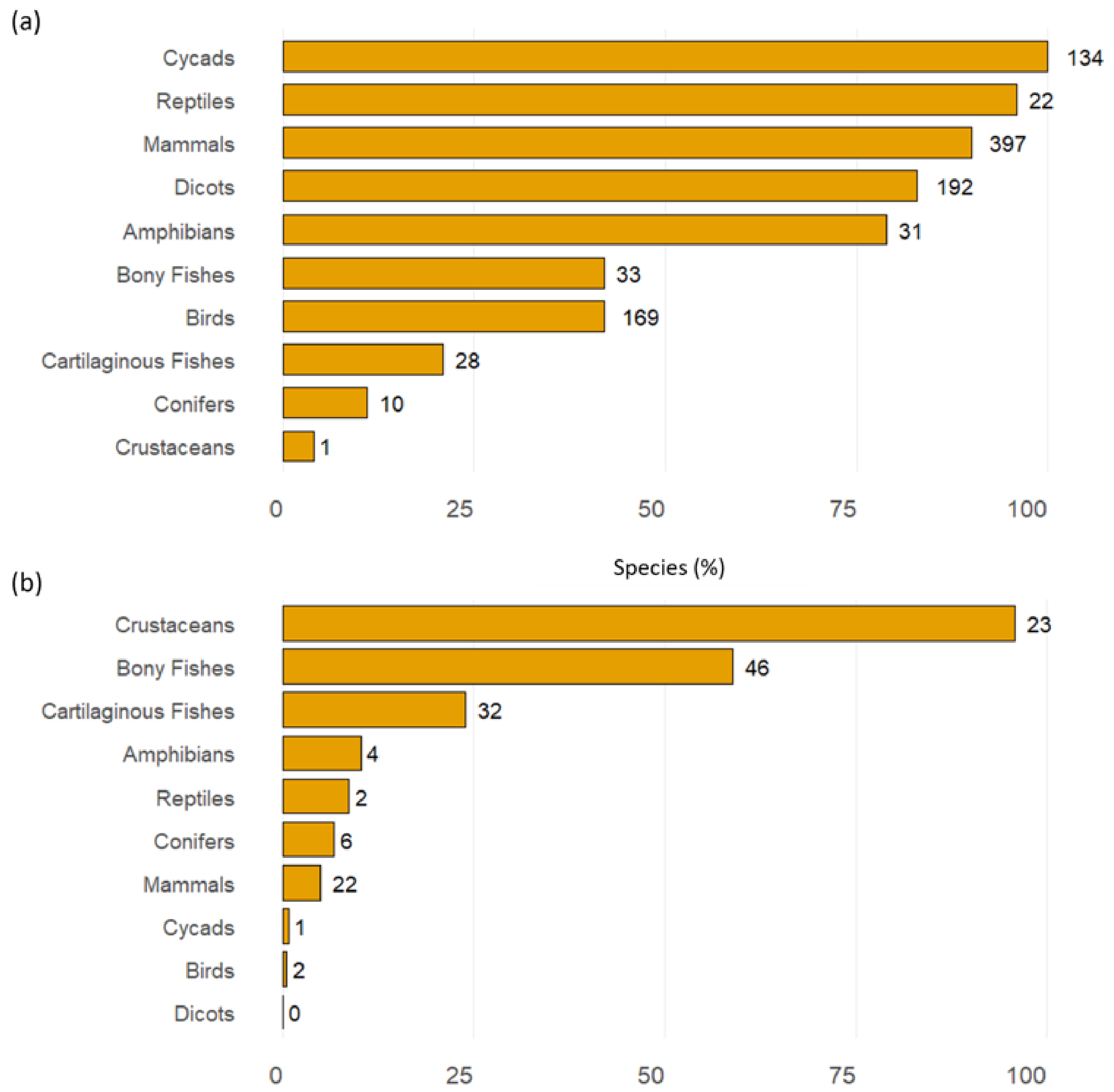

Appendix S16. Percentage of near threatened (NT) and threatened species receiving (a) international trade controls and (b) targeted species harvest management with conservation actions data documented and biological resource use (minimum) documented as a threat on the International Union for Conservation of Nature Red List. No data are available for cephalopods, cone snails or corals (Appendix S15). Bony fishes, dicotyledons (dicots) and reptiles include selected higher-level taxa (Appendix S1).

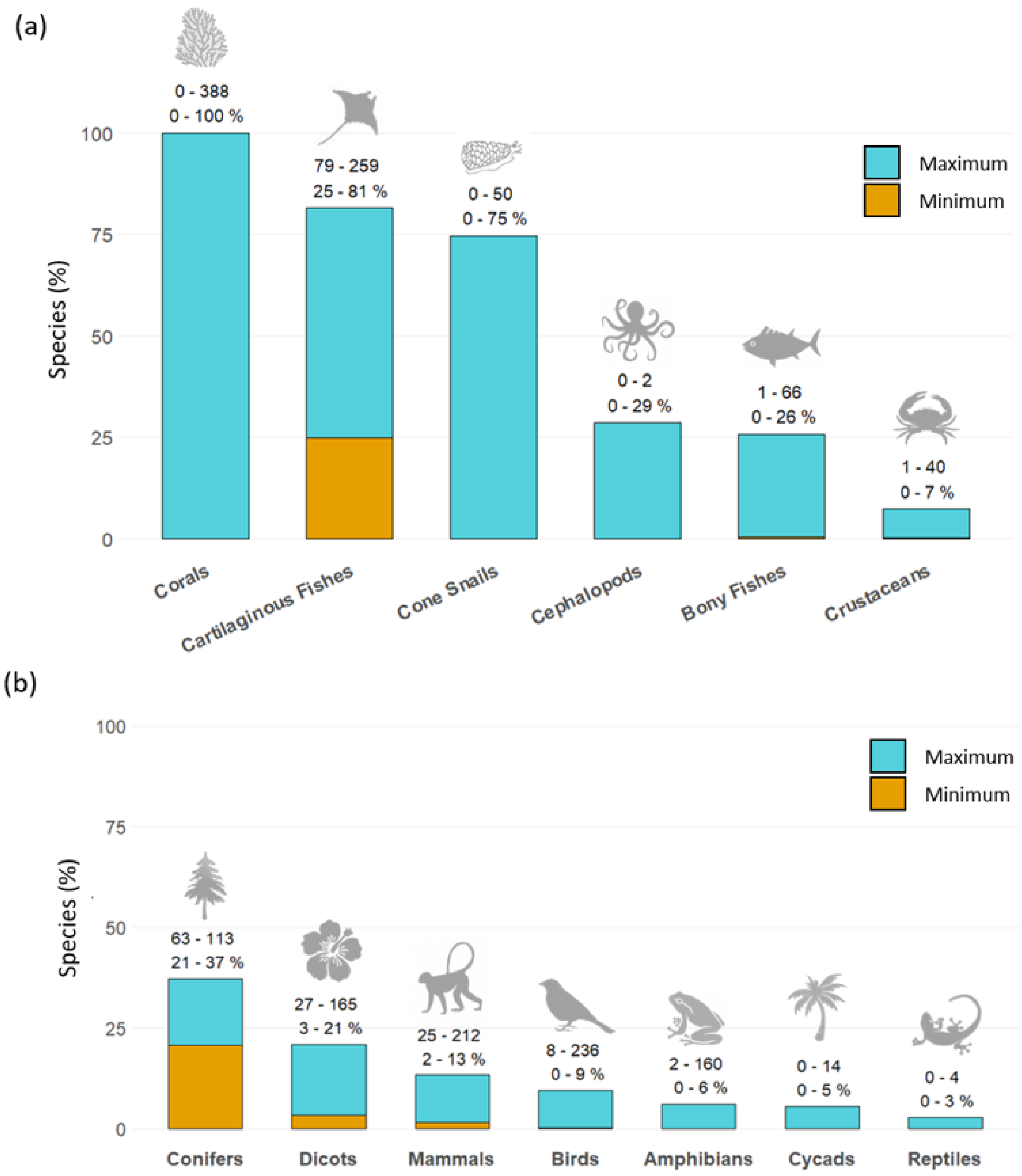

Appendix S17. Percentage of near threatened (NT) and threatened species in (a) aquatic and (b) terrestrial groups with biological resource use documented as a threat on the International Union for Conservation of Nature Red List and not receiving species conservation management

interventions to directly address that use (orange, minimum number of species not receiving either international trade control or species harvest management actions, recorded as no; blue, maximum number of species for which management actions are either not documented for the species [field is left blank], or it is not known whether the species receives those actions [recorded as unknown]; numbers above bars, range of minimum to maximum species counts and percentages within each group). Bony fishes, dicotyledons (dicots) and reptiles include selected higher-level taxa (Appendix S1).

Appendix S18. Proposed amendments to the International Union for Conservation of Nature (IUCN)'s threats classification scheme class 5.3 and 5.4 following Salafsky et al. (2008 [Appendix S2]), to account for instances where scale of use is unknown but not motivation.

Threats classification scheme 5. Biological resource use	Suggested additions*
5.3 Logging & wood harvesting	
5.3.1 Intentional use: subsistence and small scale (species being assessed is the target)	
5.3.2 Intentional use: large scale (species being assessed is the target)	
5.3.3 Intentional use: scale unknown (species being assessed is the target) [harvest]	new
5.3.4 Unintentional effects: subsistence and small scale (species being assessed is not the target)	
5.3.5 Unintentional effects: large scale (species being assessed is not the target)	
5.3.6 Unintentional effects: scale unknown (species being assessed is not the target) [harvest]	new
5.3.7 Motivation unknown or unrecorded	
5.4 Fishing & harvesting aquatic resources	
5.4.1 Intentional use: subsistence and small scale (species being assessed is the target)	
5.4.2 Intentional use: large scale (species being assessed is the target)	
5.4.3 Intentional use: scale unknown (species being assessed is the target) [harvest]	new
5.4.4 Unintentional effects: subsistence and small scale (species being assessed is not the target)	
5.4.5 Unintentional effects: large scale (species being assessed is not the target)	
5.4.6 Unintentional effects: scale unknown (species being assessed is not the target) [harvest]	new
5.4.7 Persecution and control	
5.4.8 Motivation unknown or unrecorded	

* New denotes proposed amendments to the existing classification scheme.

Appendix S19. Supplementary discussion: recommendations for improving consistency and available information in use-related Red List data

In this paper, we propose a few recommendations (Table 2) that would help to reduce the proportion of used species – currently nearly half – for which we have no evidence as to whether intentional use is sustainable or not. As discussed in the main text, we do not make these recommendations lightly, given the delicate balance between expanding the taxonomic breadth of the Red List with the best possible supporting information and the need to undertake timely reassessments and the demand on the time and resources of individual assessors. We do however consider our recommendations achievable.

Recommendation one concerns coding of the threat category motivation unknown or unrecorded. In the current threats classification scheme, if the IUCN assessor does not know the scale of harvest (whether subsistence and small scale or large scale), their only option is to select the option for motivation unknown or unrecorded under the sections 5.3 (logging & wood harvesting) and 5.4. (fishing & harvesting aquatic resources), even though they are likely to know whether the use is intentional or unintentional. For instance, we found 1,519 amphibian species for which the threat of logging and wood harvesting was recorded, of which 1,187 were recorded under motivation unknown, but the motivation must have been unintentional as these species are impacted through the loss of forest, not through direct exploitation. Although our methodology excluded these cases from our analyses, we propose a modification to the IUCN's threats classification scheme for assessors to indicate where the motivation is known, but the scale is not, to avoid these recording issues (Appendix S18).

Recommendation two is that data on timing, scope and severity of threats should be better documented as it would allow us to tease out more effectively where threat impacts are medium to high and bring greater precision to our results. For corals and cone snails, the threat of BRU is likely to be small in comparison with the impacts of bleaching and disease for corals (Carpenter et al. 2008), and urban pollution, tourism and coastal development for cone snails (Peters et al. 2013).

Further, it would be useful to better understand and quantify the degree to which species can be subject to some level of use without this resulting in them becoming threatened (i.e., impact is low, highly localized, negligible or no impact). This requires that the effects of BRU be more consistently recorded for LC species (recommendation three).

Recommendation four is for all assessments in the Red List Strategic Plan to comply with the recommended documentation requirements. One further advantage this would offer is that it would mean better availability of information on conservation actions in place and needed, respectively. Indeed, our results based on analysis of data in the IUCN's conservation actions in place classification scheme are particularly constrained because data are available for a limited number of species. For example, only one taxonomic group (conifers) has more than half of its NT and threatened species with some documentation of whether a harvest management plan is in place (as opposed to being left blank). Whether international trade controls are in place is generally better documented than whether a harvest management plan is in place. This is likely

because information on whether species are in a CITES Appendix or subject to some other policy controls is easier to obtain than on whether a harvest management plan is in place.

Finally, the addition of a check-box to indicate when the classification schemes for a given species assessment have been filled in at the recommended level, would be a powerful addition to the Red List documentation (recommendation five).
